# Microhomology-assisted scarless genome editing in human iPSCs

**DOI:** 10.1038/s41467-018-03044-y

**Published:** 2018-03-05

**Authors:** Shin-Il Kim, Tomoko Matsumoto, Harunobu Kagawa, Michiko Nakamura, Ryoko Hirohata, Ayano Ueno, Maki Ohishi, Tetsushi Sakuma, Tomoyoshi Soga, Takashi Yamamoto, Knut Woltjen

**Affiliations:** 1 Center for iPS Cell Research and Application (CiRA), Kyoto University, Kyoto, 606-8507 Japan; 2 Institute for Advanced Biosciences, Keio University, Tsuruoka, Yamagata 997-0052 Japan; 3 Department of Mathematical and Life Sciences, Graduate School of Science, Hiroshima University, Hiroshima, 739-8526 Japan; 4 Hakubi Center for Advanced Research, Kyoto University, Kyoto, 606-8501 Japan

## Abstract

Gene-edited induced pluripotent stem cells (iPSCs) provide relevant isogenic human disease models in patient-specific or healthy genetic backgrounds. Towards this end, gene targeting using antibiotic selection along with engineered point mutations remains a reliable method to enrich edited cells. Nevertheless, integrated selection markers obstruct scarless transgene-free gene editing. Here, we present a method for scarless selection marker excision using engineered microhomology-mediated end joining (MMEJ). By overlapping the homology arms of standard donor vectors, short tandem microhomologies are generated flanking the selection marker. Unique CRISPR-Cas9 protospacer sequences nested between the selection marker and engineered microhomologies are cleaved after gene targeting, engaging MMEJ and scarless excision. Moreover, when point mutations are positioned unilaterally within engineered microhomologies, both mutant and normal isogenic clones are derived simultaneously. The utility and fidelity of our method is demonstrated in human iPSCs by editing the X-linked *HPRT1* locus and biallelic modification of the autosomal *APRT* locus, eliciting disease-relevant metabolic phenotypes.

## Abstract (web summary)

Positive selection for gene targeting is a common and reliable method to generate isogenic disease models in human pluripotent stem cells. Here, the authors present engineered selection markers which achieve scarless excision by CRISPR-Cas9 and microhomology mediated end-joining.

## Introduction

Functional genomics relies on gene targeting to create or revert mutations implicated in regulating protein activity or gene expression. This methodology has advanced greatly across species through the development of designer nucleases such as ZFNs, TALENs, and CRISPR-Cas9^1,2^, with CRISPR-Cas9 taking the lead due to the simplicity of programmable sgRNA cloning, coupled with efficient and reproducible genomic cleavage. Despite differences in experimental design and DNA cleavage mechanism, all engineered nucleases function by generating targeted double strand breaks (DSBs) to induce cellular DSB repair (DSBR) pathways. Error-prone repair via non-homologous end joining (NHEJ) is typically sufficient for gene disruption, while homology directed repair (HDR) can be usurped with custom template DNA that acts as a donor in the repair of targeted double-strand breaks, allowing for more specific gene editing. These advances are of particular interest in the field of human genetics for disease modeling, where gene targeting in human induced pluripotent stem cells (iPSCs) with nucleases enables the original patient iPSC line to act as an isogenic control^3^.

Although recent advances in nuclease technology have respectably improved gene targeting efficiencies for human embryonic stem cells (ESCs) or iPSCs, the deposition of single nucleotide variations which mimic or correct patient mutations remains difficult without a robust means for enrichment and selection, such that positive selection for antibiotic resistance markers remains a staple in gene targeting^4^. Moreover, positive selection provides a method for generating clonal populations with minimal effort. For genome editing by conventional gene targeting with positive selection, scarless excision of the antibiotic selection marker is a critical step, yet remains non-trivial using current approaches. Methods such as Cre-loxP recombination^5^, and more recently excision-prone transposition^6^ have been shown to remove selection markers after their utility is expended. However, these methods are fraught with complications such as residual recombinase sites^7^, low excision frequencies, and potential for re-integration^8^. Alternative methods to achieve scarless excision must therefore be sought.

Within the repertoire of endogenous cellular repair pathways, microhomology-mediated end joining (MMEJ), is an underappreciated mechanism for repairing DSBs. MMEJ is a Ku-independent pathway that employs naturally occurring microhomology (µH) of 5–25 bp present on either side of the DSB to mediate end joining^9^. The outcome of MMEJ is a reproducible deletion of intervening sequences while retaining one copy of the µH. For this reason, MMEJ is normally considered to be mutagenic, because of an overall loss of genetic information by precise deletion.

In our current research, we address the need for high-fidelity excision by recruiting MMEJ. Using standard donor vector design where a point mutation is juxtaposed with a positive selection marker, we go on to engineer µH that flank the marker through a PCR-generated overlap in the left and right homology arms. After positive selection for gene targeting, we introduce DSBs using validated and standardized CRISPR-Cas9 protospacers nested between the selection marker and µH, stimulating the cell to employ MMEJ for scarless excision, leaving behind only the designer point mutation at the locus. Moreover, employing imperfect microhomology, we demonstrate that it is possible to produce isogenic mutant and control iPSC lines from the same experiment, addressing a current concern in the field over the effects of nuclease and cell culture manipulations^10^. We employ this technique in human iPSCs to edit hypoxanthine phosphorybosyltransferase 1 (*HPRT1*) and biallelically edit adenosine phosphoribosyl transferase (*APRT*), deriving iPSC models and isogenic controls for HPRT_Munich_ partial enzyme deficiency^11^ and the common Japanese APRT*J allele^12^, respectively. Measures of cellular metabolism establish consistent disease phenotypes between engineered iPSC clones, as compared to concordant controls. We expect this technique to have broad applications, even beyond scarless iPSC genome editing.

## Results

### MMEJ bias towards precise repair outcomes

**Fig. 1:**
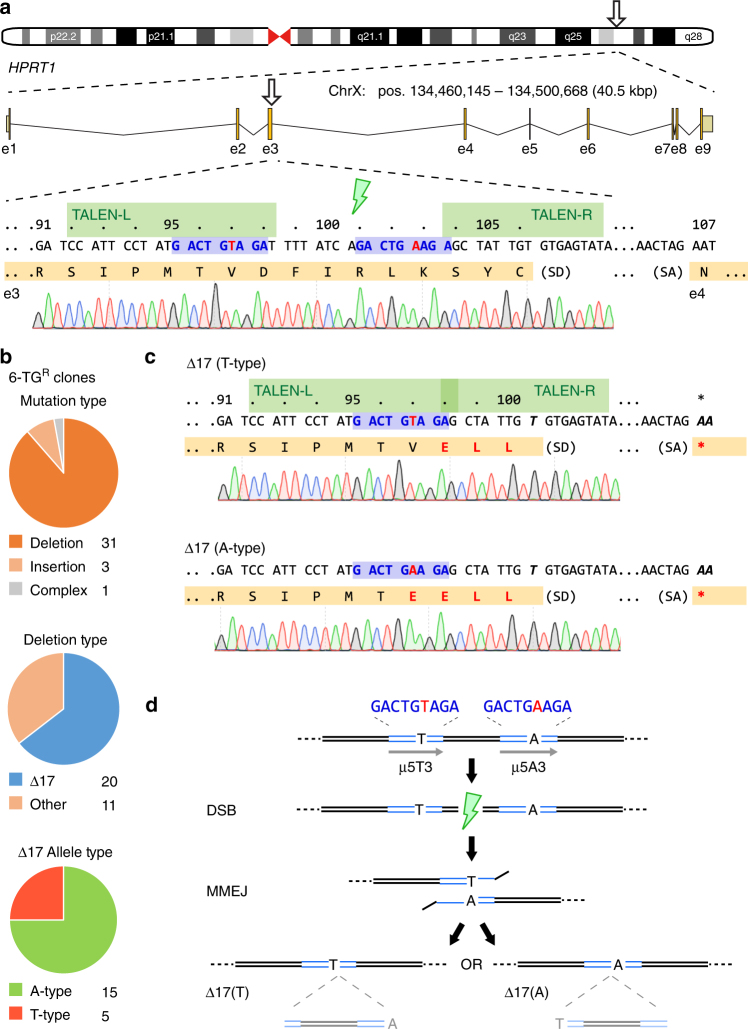
TALEN disruption of the *HPRT1* locus is biased by endogenous microhomology. **a** Schematic of the human *HPRT1* locus with detail for segments of exon 3 and 4 (orange) including splice junctions, the *HPRT1*_B NC- or Avr-TALEN target sites (green), and predicted μ5W3 microhomology (blue) with the mismatched base (A/T) shown in red. Chromosome positions refer to *H. sapiens* GRCh38. *HPRT1* codons are numbered above. 1383D6 iPSC sequence trace genome is shown below. SD, splice donor; SA, splice acceptor. **b** Summary of repair outcomes in 6-TG^R^ clones following transfection of 1383D6 iPSCs with *HPRT1*_B Avr-TALENs. Individual clone sequences are listed in Supplementary Fig. 5. **c** Sequence of the two most commonly observed 17 bp deletions. **d **Schematic of the molecular repair events leading to either Δ17(A) or Δ17(T) formation by MMEJ. Note that the intervening 17 bp sequence is similarly excised, despite the final outcome (A or T). μH, microhomology (blue)

Gene disruption using programmed endonucleases relies on cellular error-prone repair pathways such as NHEJ to produce out-of-frame insertion and deletion (indel) mutations. We previously exploited this phenomenon to disrupt HPRT enzyme function in 201B7 human female iPSCs in order to assess the activities of modified TALEN architectures^13^. In that assay, transient transfection of TALENs modeled after *HPRT1*_B^14^ which target exon 3 of the human *HPRT1* gene (Fig. 1a), followed by metabolic enrichment for HPRT loss-of-function by 6-thioguanine resistance (6-TG^R^; Supplementary Fig. 1) revealed a recurring mutation comprised of 17 deleted bases (Δ17). TALEN-mediated disruption of HPRT1 in another female iPSC line (409B2) reproduced the Δ17 allele at a frequency of ~12% (Supplementary Fig. 2). DSBR outcomes may be biased by short direct sequence repeats towards alternative MMEJ repair^9^. We therefore used a modified script^15^ to detect µH at the expected DSB site and identified a 5 bp µH (µ5: ‘GACTG’) lying within the left TALEN (TALEN-L) binding site and the intervening spacer region, separated by heterologous sequence (Fig. 1a). Further examination revealed a shorter µH of 3 bp (µ3: ‘AGA’) adjacent to µ5, separated by only one variant base (T or A), resulting in an imperfect compound µH of the structure ‘GACTGWAGA’, where W = T/A (hereafter referred to as µ5W3). These observations suggested a biased repair pathway through MMEJ which warranted further investigation.

Considering the *HPRT1* locus is X-linked, we set out to explore the spectrum of DSBR outcomes induced by TALEN in male 1383D6 iPSCs^16^ and H1 ESCs^17^. Whilst maintaining the same target sequences^14^, *HPRT1*_B TALENs were updated from a truncated *Xanthomonas oryzae* pv. (PthXo1)-based TALE scaffold^13^ to *X. campestris* pv. *vesicatoria* (AvrBs3)-based +136/+63 TALE architecture^18^ and expressed from a CAG promoter-driven expression vector. These TALEN vector modifications resulted in a 3-fold increase in cleavage activity for Avr*HPRT1_B* TALENs as measured by single-strand-annealing (SSA) assay^19^ (Supplementary Fig. 3a). Enhanced genome cleavage activity was also demonstrated in 1383D6 male iPSCs by improved *HPRT1* knockout as measured by 6-TG^R^ colony formation (Supplementary Fig. 3b). We estimated allele frequencies in a bulk population of 6-TG^R^ iPSCs by employing computational sequence trace decomposition (TIDE) from mixed PCR amplicons^20^. In the sequence trace file, overlapping peaks were observed immediately following µ5W3, with a preceding T/A overlay at position ‘W’ (Supplementary Fig. 4a-c). Amongst a variety of minor deletion alleles, Δ17 was found to be significantly overrepresented (63.5%, Supplementary Fig. 4d), strongly supporting MMEJ through µ5W3. TIDE verified a similar frequency in male H1 human ESCs (43.9%, Supplementary Fig. 4e-g). In order to exclude the possibility that this apparently high rate of MMEJ repair in the population was an artifact of PCR bias, we isolated 6-TG^R^ iPSC clones and performed Sanger sequencing of exon 3 (Supplementary Fig. 5). Clonal analysis revealed deletions as the most common DSBR outcome (~88%), amongst which the Δ17 allele comprised the majority (~64%), consistent with the population-based TIDE analysis. The Δ17 alleles could be further subdivided according to the imperfection in µ5W3 at a ratio of 5(T):15(A) (Fig. 1b,c), presumably dictated by more frequent use of the longer µ5 for repair, and a concordant loss of the intervening ‘TAGA’ sequence. Both ∆17 deletion types produce a -1 frame shift which results in three (D98E, F99L, I100L, for *HPRT*^*∆17T*^) or four (V97E, D98E, F99L, I100L, for *HPRT*^*∆17A*^) missense mutations terminating in a nonsense mutation (*fsTer101*), resulting in loss of HPRT function as measured by resistance to 6-TG and sensitivity to HAT (Supplementary Fig. 6), with no additional effects on clone morphology or growth rate under normal culture conditions.

Analysis of the TALEN-mediated *HPRT1* knockout data led us to two key conclusions (Fig. 1d): first, that common MMEJ events result in high-fidelity deletion of intervening sequence, and second, that MMEJ between imperfect µH (µ5W3) leads to alternate yet predictable allelic outcomes.

### Coincident editing of mutants and isogenic controls

**Fig. 2:**
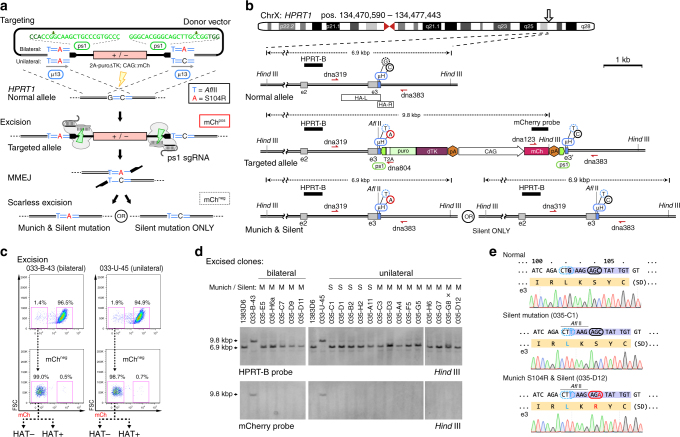
Imperfect microhomology simultaneously creates iPSCs with patient mutations and their isogenic controls. **a** Schematic overview of the MMEJ method for editing HPRT_Munich_ and control alleles. Left and right homology arms overlap, generating a 13 bp tandem µH (blue) flanking the selection cassette (red). The patient mutation (c.312C > A, red) is present in one µH (unilateral) or both (bilateral). A silent bilateral point mutation (c.306G > T, blue) generates an *Afl*II site. Complementary ps1 protospacers (black) are nested divergently between the µH and cassette, with sequences and cut site positions indicated in green above. Gene targeting used Avr*HPRT1_B* TALENs (yellow bolt). Upon transfection of targeted clones with CRISPR-Cas9 (pX-ps1), DSBs are generated flanking the cassette, proximal to the engineered µH (green bolts). Repair by MMEJ scarlessly excises the cassette, resulting in two possible editing outcomes. **b** Detailed schematic of *HPRT1* gene targeting and MMEJ resolution. Exons (gray), overlapping homology arms (HA-L/R, white), µH (blue), ps1 CRISPR-Cas9 target sites (green), and engineered bases are indicated. 2A-puroΔTK is inserted in-frame with *HPRT1* exon3. Black bars indicate Southern blot probes for the indicated restriction fragments. Genotyping primers are shown in red. **c** FACS scheme used to enrich mCh^neg^ cassette-excised iPSCs. Sorted populations were plated with or without HAT selection for clonal analysis to determine the frequency and fidelity of repair. **d** Southern blot analysis of select excised clones revealing restoration of the *HPRT1* locus (HPRT-B probe, top) and removal of the cassette (mCherry probe, bottom). Parental 1383D6 and intermediate 033-U-45 and 033-B-43 targeted iPSCs are included as controls. Genotypes (S, Silent only; M, Munich and Silent) are indicated above. “x” indicates one clone with aberrant banding. **e** Sequencing examples of iPSC clones with Munich and/or Silent mutations derived from clone 033-U-45 (unilateral). Both types of clones are isolated from the same excision experiment

Genome targeting in human iPSCs benefits from antibiotic enrichment, yet to achieve scarless editing of patient mutations selection markers must be removed entirely^21^. Inspired by TALEN-mediated *HPRT1* disruption by MMEJ (Fig. 1d), we reasoned that by engineering duplicated µH from unique endogenous sequences, such that they flank the antibiotic selection marker, we could induce the cell to employ MMEJ to repair nested DSBs resulting in scarless excision and locus restoration with only engineered point mutations remaining (Fig. 2a). To demonstrate this microhomology-assisted excision (MhAX) technique, we chose to edit bases within exon 3 of *HPRT1*. As *HPRT1* is expressed in human iPSCs, we employed a puro∆TK (a fusion of puromycin to truncated thymidine kinase) antibiotic counter-selection marker as a 2A-peptide linked promoterless gene-trap; an approach similar to that used for background-free AAVS1 targeting^16^, but lacking a splice-acceptor sequence in favor of in-frame insertion into the *HPRT1* open reading frame (Fig. 2b). As well, we included a constitutively expressed CAG::mCherry reporter gene with the intent to track both gene targeting and excision steps. In order to introduce DSBs flanking the selection/reporter cassette, we chose to employ CRISPR-Cas9 rather than TALEN, exploiting multiple advantages including: a unified Cas9 protein and sgRNA plasmid expression system^22^ and defined endonuclease breakpoints^23^. In selecting protospacer/sgRNA combinations, we focused on three sgRNAs targeting the GFP gene of *A.victoria*, already shown to have high activity and low toxicity in human U2OS osteosarcoma cells^24^. Activity of each GFP sgRNA was determined using an SSA assay in HEK293T cells (Supplementary Fig. 7a and b), and an AAVS1-CAG::eGFP disruption assay in human iPSC (Supplementary Fig. 7c). No overt cytotoxicity was observed for any of the sgRNAs in either assay. Based on these data, we selected the eGFP1 protospacer (hereafter referred to as ps1). Duplicated ps1 protospacers were positioned flanking the cassette in a divergent orientation such that the PAMs and upstream cleavage sites were proximal to the engineered µH (Fig. 2a). High-throughput screening and computational analysis of sgRNA libraries^25^ has revealed that a ‘G’ nucleotide positioned downstream of the PAM is unfavorable for Cas9 activity. We therefore defined potential µH in the genome such that each nested ps1 PAM would be flanked by a ‘T’ or an ‘A’ nucleotide (Supplementary Table 1).

Considering our observations for repair of the imperfect µ5W3 at the *HPRT1* locus (Fig. 1), we surmised that the duality of outcomes could be intentionally exploited to produce both mutant and control iPSC clones from a single experiment. We chose to focus on re-creating the HPRT_Munich_ partial enzyme deficiency^11^, originally discovered in a patient presenting with gout caused by hyperuricemia. The HPRT_Munich_ allele results from a C-to-A transversion mutation (rs137852485; c.312C>A; S104R)^26^ located within exon 3 of *HPRT1* neighboring the Avr*HPRT1_*B TALEN target site. Through an overlap in *HPRT1* homology arms, we created a flanking µH ‘TAAGAG**A**TATTGT’ which contained the Munich c.312C>A mutation centrally (bold underlined) and an additional Silent c.306G>T mutation at the 5’ end of the µH (underlined) that generated an *Afl*II restriction site exclusively for diagnostic purposes (Fig. 2a and Supplementary Table 1). In order to recapitulate the imperfect µH phenomenon, we generated two targeting vectors in which the Munich c.312A patient mutation was either symmetrical (bilateral), or asymmetrical (unilateral, such that the downstream homology is ‘TAAGAGCTATTGT’) (Fig. 2a). Bilaterally encoded mutations were hypothesized to be deposited in 100% of clones, while unilaterally encoded mutations would be deposited in only a fraction of clones. Both µH contained the diagnostic *Afl*II c.306G>T mutation.

Avr*HPRT1*_B TALENs were employed to stimulate gene targeting in 1383D6 iPSCs. Puro^R^ clones were screened by Southern blot genotyping, mCherry expression by FACS, sensitivity to HAT, and resistance to 6-TG (Supplementary Fig. 8a and 9a,b). Excision was induced by transfection with pX-ps1, producing mCh^neg^ populations at a rate of 1.4 and 1.9% for 033-B-43 (bilateral) and 033-U-45 (unilateral), respectively (Fig. 2c). mCh^neg^ cells were therefore FACS sorted to >98% purity and replated at clonal density with or without HAT selective pressure. Under HAT selection 033-B-43 yielded no clones, suggesting either a failure to repair or a phenotypic effect of the Munich c.312A mutation (Table 1). On the other hand, under HAT selective pressure 033-U-45 generated iPSC colonies which all achieved scarless excision but represented deposition of the Silent c.306T mutation exclusively (49/49), indicating either a repair bias or supporting the possibility that HPRT_Munich_ clones retain sensitivity to HAT.

**Table 1:** Rates of scarless editing at the HPRT locus using engineered microhomology

Parent clone	Munich mutation	Enrichment	Samples analyzed	Normal allele	NHEJ (perfect)	Scarless excision^a^	Fidelity (%)^b^	Silent ONLY	Munich & Silent
033-B-43	bilateral	HAT	0	n/a	n/a	n/a	n/a	n/a	n/a
		no HAT	179	0	171 (75)	8	4.5	0	8
033-U-45	unilateral	HAT	49	0	0	49	100	49	0
		no HAT	206	0	192 (84)	14	6.8	5	9

^a^repair events without any additional base mutations, as predicted to occur via MMEJ

^b^calculated as (Scarless excision/Samples analyzed)

Scarlessly engineered HPRT_Munich_ alleles were produced in the absence of HAT selective pressure (Table 1). Southern blotting of clones (Fig. 2d) provided evidence that the HPRT locus was reconstituted and that transgenes had not re-inserted into the genome at any detectable rate. Releasing HAT selective pressure also revealed clones that repaired via NHEJ (Table 1), many of which (>40%) represented perfect end-joining comprised of ps1 breakpoints, PAMs, and retention of flanking µH (Supplementary Fig. 8b). From parental clones with bilateral µH, 4.5% (8/179) excised scarlessly, and all clones bore both the Silent c.306T and Munich c.312A mutations. Clones from unilateral µH excised scarlessly at a rate of 6.8% (14/206). Importantly, 9/14 clones bore both the Silent and Munich mutations, while the remainder (5/14) carried only the Silent mutation (Table 1) as verified by sequencing (Fig. 2e) and restriction fragment length polymorphism (RFLP) analysis (Supplementary Fig. 8c), indicating that we could reproduce the stochasticity of MMEJ outcomes by intentionally engineering imperfect homology.

Finally, we set out to examine the phenotypic consequences of HPRT editing and assess clonal variation. Under normal iPSC maintenance conditions, no difference in morphology or growth rate was noted between normal, mutant, or isogenic control clones (Supplementary Fig. 9a and b). Within 24 h of HAT treatment knockout iPSCs were completely eliminated, while HPRT_Munich_ iPSCs showed delayed growth by cell number and a profound change in cell morphology (Supplementary Fig. 9b, bottom), leading to complete cell death by prolonged treatment. Interestingly, unlike knockout iPSCs, HPRT_Munich_ iPSCs also retained a delayed sensitivity to 6-TG (20 µM, Supplementary Fig. 9a). Previously, in vitro assays using HPRT_Munich_ patient cell lysates indicated abnormal hypoxanthine catalytic activity^27^ although protein levels were normal^11,28^. Accordingly, while HPRT protein was undetectable in Western blot analysis of knockout iPSC line lysates, HPRT_Silent_ or HPRT_Munich_ clones revealed normal protein expression levels (Supplementary Fig. 9c). Pathologically, excess hypoxanthine is converted into uric acid (Supplementary Fig. 1) which can accumulate in the joints and tendons causing inflammatory arthritis, kidney stones, or urate nephropathy. Capillary-electrophoresis mass spectrometry (CE-MS) was used to detect ionic metabolites in spent cell culture media^29,30^. While HPRT_Silent_ clones had metabolic profiles resembling 1383D6, HPRT_Munich_ iPSCs accumulated both hypoxanthine and guanine, but to a lesser extent than Δ17 or 033-U-45 knockouts (Supplementary Fig. 9d). These cell growth and metabolome data are consistent with a low-level salvage of guanine and hypoxanthine in HPRT_Munich_ cells which is insufficient for DNA replication and growth in the absence of de novo synthesis. As such, we generated a unique iPSC model of an *HPRT1* coding-region variant, using the MhAX technique to scarlessly and stochastically deposit disease-relevant or control point mutations.

### Parameters affecting MMEJ cassette excision

**Fig. 3:**
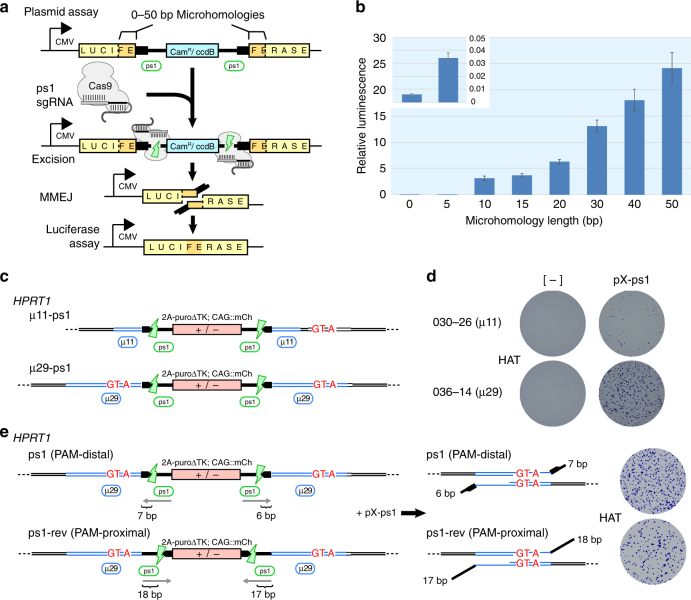
Parameters affecting cassette excision by MMEJ. **a** Schematic of the plasmid-based MMEJ assay measuring luciferase repair. **b** Luciferase activity as a function of increasing flanking μH length. Inset shows luciferase activity with 5 bp μH compared to background (0 bp). Error bars show s.e.m. (*n* = 3). **c** Schematic of the *HPRT1* chromosomal assay depicting MhAX cassettes and nested ps1 protospacers with flanking 11 or 29 bp of μH. Synonymous mutations are shown in red. **d** HAT^R^ colonies arising from targeted clones without or with nuclease (pX-ps1) transfection. One representative clone is shown for each homology length. **e** Schematic of *HPRT1*-targeted μ29 MhAX cassettes with inverted ps1 protospacers. Predicted heterology lengths are indicated for each DSB. HAT-resistant colonies following excision are representative of three independent experiments. HAT-selected populations from either protospacer orientation are enriched for MMEJ repair (Supplementary Fig. 12c)

In order to explore the effects of increasing µH length on MMEJ efficiencies^31^, we developed a plasmid-based MMEJ assay analogous to our cassette design used to generate the HPRT_Munich_ allele. We flanked a Cam^R^/*ccdB* positive/negative bacterial selection marker with ps1 protospacers and inserted it into a luciferase expression vector with flanking µH of increasing length from 0—50 bp (Fig. 3a,b). Following transfection into HEK293T cells, a positive correlation between µH length and luciferase activity was observed, suggesting an improved rate of MMEJ (Fig. 3b).

Excision from an extrachromosomal plasmid in HEK293T cells may not accurately reflect precise excision from the iPSC genome. We therefore established a contextually relevant chromosomal assay at the HPRT locus where cassette excision by MMEJ results in recovery of HAT resistance, along with the deposition of three synonymous mutations disrupting µ5A3 (c.303A>G, c.304C>T, and c.306G>A) (Supplementary Fig. 10 and 11). Using TALEN, MhAX cassettes flanked by µH of 11 bp ‘TGACTGTAGAT’, or 29 bp ‘TGACTGTAGATTTTATCAGGTTAAAGAGC’, the latter of which contained the synonymous mutations (underlined, Supplementary Table 1), and with nested ps1 protospacers were targeted to *HPRT1* exon 3 (Fig. 3c). Excision using µ29 gave rise to higher numbers of HAT^R^ colonies (Fig. 3d) even though mCh^neg^ fractions were similar between the two constructs (Table 2 and Supplementary Fig. 12a), indicating that Cas9 cleavage and cassette excision rates were not affected by µH length but rather led to enhanced scarless repair by MMEJ. Genotyping of *HPRT1* alleles from µ11 and µ29 mCh^neg^ populations (without HAT enrichment) revealed a >4-fold increase in scarless repair and mutation deposition (7.8% vs ~35% avg.), similar to the fold-change observed in the plasmid assay (Fig. 3b). Thus, increasing the length of µH improves scarless excision from human iPSC chromosomes.

**Table 2:** Microhomology length affects MMEJ repair of human chromosomes

Parent clone	µH (bp)	Excision (% mCh^neg^)	Scarless excision	Fidelity (%)
030-26	11	3.5	6/77	7.8
036-08	29	2.8	17/45	35.6
036-12	29	3.4	29/82	35.4
036-14	29	2.8	16/53	30.2

Evidence from DSBR in yeast^32^ and mouse ESCs^33^ suggests that the presence of long heterology (non-homologous sequence from the end of DSBs until the start of homology) can negatively impact MMEJ or HDR repair rates. We tested this parameter by inverting the ps1 protospacers (ps1-rev), such that their PAMs were placed proximal to the cassette, leading to a 17 or 18 bp heterology on either end compared to 6 or 7 bp generated in the PAM-distal orientation used thus far (Fig. 3e and Supplementary Table 1). Cassette excision rates (% mCh^neg^) using PAM-distal or -proximal protospacers were similar (Supplementary Fig. 12b), indicating that orientation itself does not affect CRISPR-Cas9 cleavage. However, MMEJ repair rates were impeded by elongated heterology as indicated by a reduction in overall HAT^R^ colony formation following excision of ps1-rev alleles (Fig. 3e, right). Based on these results, subsequent MhAX experiments employed elongated µH and maintained ps1 in a PAM-distal orientation for reduced heterology.

### Biallelic modification of the *APRT* locus

**Fig. 4:**
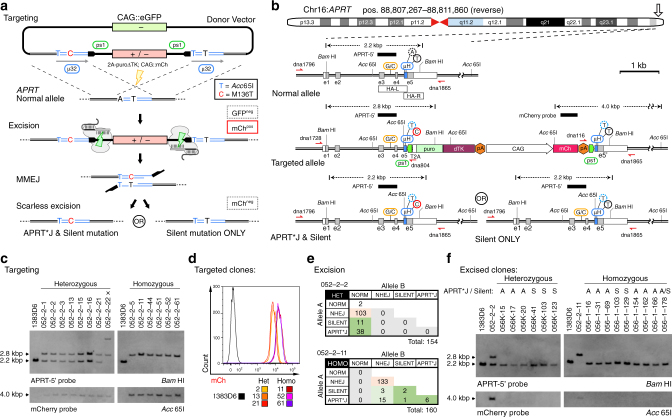
Biallelic modification of the *APRT* locus. **a** Schematic overview of the method for scarless engineering of APRT*J and control alleles. Homology arm overlap generates a 32 bp tandem µH (blue), with the patient mutation (c.407T > C, red) present unilaterally, and the Silent mutation (c.402A > T, blue) present bilaterally. A GFP reporter is included in the backbone to exclude cells with random donor integration by FACS. Gene targeting used CRISPR-Cas9 (yellow bolt, Supplementary Fig. 13). The remaining elements are as described in Fig. 2a. **b** Detailed schematic of *APRT* gene targeting and MMEJ resolution. The heterozygous SNP (rs8191489) is shown in orange. Additional labeling is consistent with Fig. 2b. **c** Southern blot analysis of select *APRT* hetero- and homozygously targeted clones using genomic (APRT-5’, top) and transgenic (mCherry, bottom) probes. Parental 1383D6 iPSCs are included as a control. “x” indicates one clone with aberrant banding. **d** Histograms of mCherry fluorescence intensities in select *APRT* targeted clones. Note that the bimodal distribution is correlated with genotype, and therefore CAG::mCh transgene copy-number. **e** *APRT* diploid genotypes of clones. Heterozygous genotypes were resolved using TIDE. Alleles marked as ‘APRT*J’ were also edited with the Silent mutation. **f** Southern blot analysis of select excised clones revealing restoration of the *APRT* locus (APRT-5’ probe, top) and removal of the cassette (mCherry probe, bottom). Parental 1383D6 and intermediate targeted iPSCs (from **c**) are included as controls. Genotypes (S, Silent only; A, APRT*J and Silent) are indicated above

Many disease-causing mutations show autosomal recessive inheritance. To demonstrate scarless biallelic modification using the MhAX method, we chose to edit the adenosine phosphorybosyl transferase (*APRT*) gene, which produces the enzyme required for the synthesis of adenosine monophosphate (AMP) from adenine (Supplementary Fig. 1). The APRT*J allele (rs104894507; c.407T>C; M136T) results in partial enzyme deficiency causing a buildup of 2,8-dihydroxyadenine (2,8-DHA) crystals, often leading to kidney stone formation or more severely, kidney failure^12^. Although APRT*J is prevalent in Japanese patients with urolithiasis (79%), an in vitro iPSC model remains to be generated. Employing a gene-trap selection marker and constitutive reporter cassette flanked by PAM-distal ps1 protospacers, we engineered a flanking 32 bp µH (GTACCA**C**GAACGCTGCCTGTGAGCTGCTGGGC) in which a synonymous c.402A>T Silent mutation (underlined) generating a diagnostic *Acc*65I restriction site was present bilaterally, while the c.407T>C APRT*J mutation (bold underlined) was present unilaterally (Fig. 4a and Supplementary Table 1). CRISPR-Cas9 sgRNAs overlapping the mutation sites in *APRT* exon 5 were screened using T7EI digestion and directly in iPSCs by *APRT* gene targeting (Supplementary Fig. 13). APRT sgRNA-2 was selected for superior performance in both assays. In order to reduce random integration of the donor vector backbone, we employed negative selection for GFP fluorescence^34^ (Fig. 4a). Puro^R^, mCh^pos^/GFP^neg^ iPSC clones were identified by microscopy, picked, and genotyped (Fig. 4b,c). Mean mCherry fluorescence intensity displayed a bimodal distribution (Fig. 4d), which was linked to copy number by genotyping heterogously and homozygously targeted clones.

Three each of hetero- and homozygously targeted clones were subjected to cassette excision via transfection of pX-ps1. Excision rates were consistently higher for heterozygous (6.7% avg.) versus homozygous (3.3% avg) targeted clones (Table 3 and Supplementary Fig. 14), reflecting the requirement for one or two copies of the cassette to be removed from the genome. Excised mCh^neg^ populations were isolated by FACS, from which the spectrum of alleles was analyzed by Sanger sequencing of genomic PCR products (Table 3). Expectedly, approximately half of the sequences detected in excised populations from heterozygous targeted clones were unmodified normal alleles. Scarless excision of the cassette occurred at an average rate of 30% amongst heterozygous clones. Homozygous targeted clones showed an overall reduced rate of scarless excision (13% avg.), lending to a relative increase in NHEJ alleles. Co-deposition of the Silent and APRT*J mutations was more common than Silent alone, possibly due to the unbalanced nature of the imperfect µH (µ6Y25; Supplementary Table 1). Thus, unilateral µH was again observed to stochastically generate both silent and pathogenic allele types.

**Table 3:** Rates of scarless editing at the *APRT* locus using engineered microhomology

Parent clone	Genotype	Excision (%mCh^neg^)	Samples analyzed	Normal allele	NHEJ (perfect)	Scarless excision^a^	Fidelity (%)^b^	Silent ONLY	APRT*J & Silent
052-2-2	Het	8.4	37	23	9 (5)	5	35.7	1	4
052-2-13	Het	5.0	50	30	17 (8)	3	15.0	1	2
052-2-21	Het	6.8	46	24	14 (11)	8	36.4	4	4
052-2-11	Homo	3.4	45	0	40 (26)	5	11.1	0	5
052-2-52	Homo	3.6	53	0	47 (30)	6	11.3	1	5
052-2-61	Homo	2.9	46	0	38 (30)	8	17.4	0	8

^a^Repair events without any additional base mutations, as predicted to occur via MMEJ

^b^Calculated as (Scarless excision/Samples analyzed)

From populations of mCh^neg^ cells, clones were isolated and genotyped. To ensure the identification of both alleles, we included a neighboring heterozygous SNP (rs8191489, G/C) from intron 3 within the PCR amplicon (Fig. 4b and Supplementary Fig. 15a), and employed TIDE analysis to decompose heterozygous repair events. The diploid genotypes of all clonally isolated iPSCs are summarized in Fig. 4e. Scarless excision rates in the heterozygously targeted clone 052–2–2 were 31.8% (49/154 clones), similar to that predicted from population analyses (Table 3). Homozygous clone 052–2–11 gave rise to 5.6% (9/160 clones) with scarless biallelic modification, representing homozygous and compound heterozygous edited genotypes (Fig. 4e and Supplementary Fig. 15a). Sequence decomposition revealed that an additional 18 clones with one NHEJ allele underwent scarless excision of the other allele (Supplementary Fig. 16), such that the frequency of clones having at least one scarlessly edited allele (27/160 clones, or 16.9%) was in agreement with our initial population analysis.

Monoallelically and biallelically edited iPSC clones were selected and correct gene editing was further confirmed using Southern blot (Fig. 4f) and an *Acc*65I RFLP assay (Supplementary Fig. 17). We phenotyped edited clones by testing their resistance to 2,6-diaminopurine (DAP, Supplementary Fig. 1 and 15b), a toxic purine analog^35^. Parental 1383D6 and homozygous *APRT*^*Silent/Silent*^ mutants displayed severe drug sensitivity to 10 µg/mL DAP treatment, with nearly complete cell killing within just 48 h (Supplementary Fig. 15b). Heterozygous targeted or APRT^*J/Silent^ cells had reduced sensitivity to DAP but were essentially eliminated within 48 h, while homozygous targeted and APRT^*J/*J^ cells were completely resistant to DAP treatment. This data verifies a reproducible change in cellular metabolism amongst *APRT* gene-edited iPSCs.

### Expedited generation of an isogenic allelic series

**Fig. 5:**
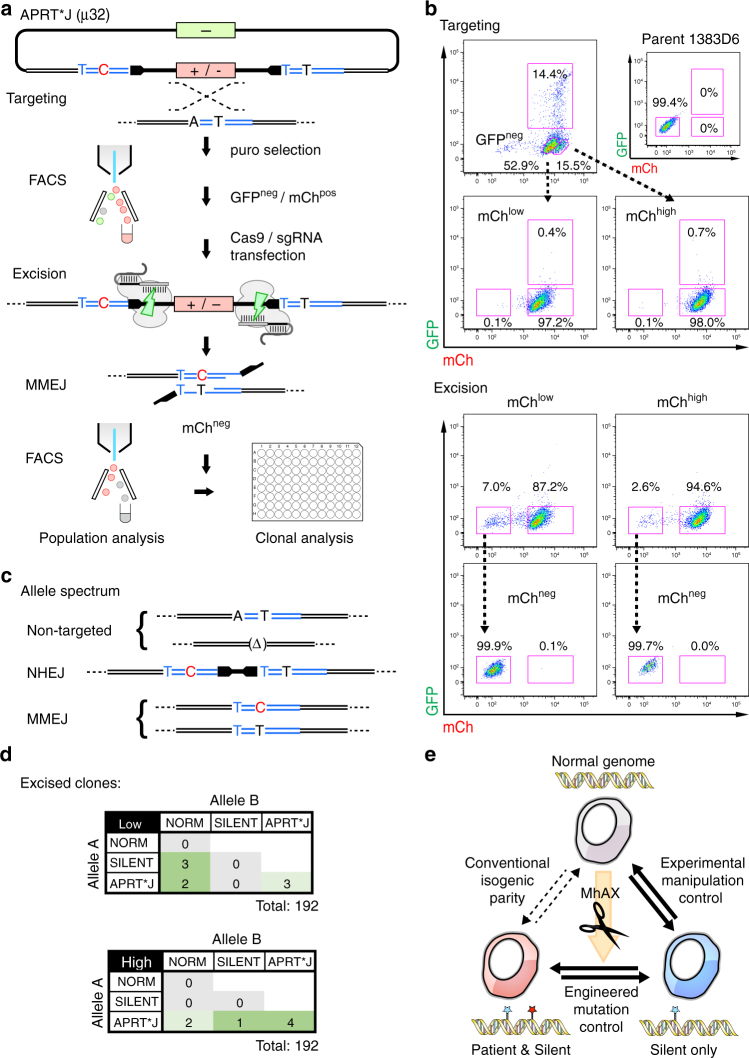
Expedited biallelic gene editing by FACS sorting. **a** Schematic of the FACS sorting protocol. GFP^neg^ / mCh^pos^ cells (targeted) are isolated in bulk and subjected to nuclease transfection, followed by sorting populations for mCh^neg^ (excised). Resolved alleles were screened in the population, or in single-cell derived iPSC clones. The donor vector, allele and additional features are as described in Fig. 4a,b. **b** Representative FACS plot for the targeting and excision steps. GFP^pos^ cells were excluded, and mCh^pos^ cells were divided into high and low fractions to bias monoallelically and biallelically targeted cells, respectively. **c** Predicted *APRT* allele spectrum within the sorted mCh^neg^ populations. **d** *APRT* diploid genotypes of clones. Only clones with Normal or MMEJ-resolved alleles are shown. Alleles marked as ‘APRT*J’ were also edited with the Silent mutation. **e** By employing imperfect μH, MhAX derives edited cells and their concordant isogenic controls simultaneously, reducing technical variation

With the goal of expediting the scarless gene editing process in iPSCs, we chose to exploit the high fidelity of gene-trap targeting with copy-number dependent transgene expression and fluorescent counter-selection of random targeting events by FACS (Fig. 5a). *APRT* gene targeting was carried out as described above (Fig. 4), however instead of clonal isolation and screening of targeted intermediates, entire puro^R^ populations were harvested in bulk and subjected to FACS to isolate mCh^pos^/GFP^neg^ iPSCs (Fig. 5a,b). We further separated the mCh^pos^ population into mCh^low^ (52.9% of total) and mCh^high^ (15.5% of total) (Fig. 5b) in order to enrich for heterozygous or homozygously targeted cells (Fig. 4d), respectively. Cassette excision was more efficient from the mCh^low^ than mCh^high^ (7.0 vs 2.6%) bulk population (Fig. 5b), consistent with excision one or two transgene copies from heterozygous or homozygously targeted clones (Table 3), suggesting that the MhAX method may be expedited by FACS when the fidelity of targeting is high.

Genotyping of the two excised populations classified alleles into 3 categories: non-targeted, which includes normal alleles or indel alleles generated by APRT sgRNA-2 during gene targeting; NHEJ, which arise during repair of cassette excision (distinguished from APRT sgRNA-2 indels as they retain engineered µH and cassette sequences, similar to that shown in Supplementary Fig. 8b); and MMEJ, which resolve scarlessly and retain the pathogenic APRT*J and/or Silent mutations (Fig. 5c and Table 4). Notably, while the mCh^high^ population was biased toward NHEJ and MMEJ, the mCh^low^ population contained more frequent indels (37.5 vs 6.1% for mCh^high^), validating FACS enrichment of monoallelically or biallelically targeted cells, but also reflecting the potential of CRISPR-Cas9 to elicit error-prone repair of DSBs in opposition to HDR. A similar process of FACS-based targeting and excision for the X-linked HPRT_Munich_ allele (Supplementary Fig. 18 and Supplementary Table 2) gave rise to scarless gene edited clones at a rate comparable to that observed previously for cloned intermediates (5.6 vs 8%; Table 1) without a significant proportion of normal or indel alleles. Excluding non-targeted normal and indel alleles from the *APRT* analysis, the fidelity of scarless repair of target alleles was estimated to be slightly higher for the mCh^low^ population compared to mCh^high^ (26.5 vs 22.7%). Finally, we performed clonal isolation from bulk excised populations for the analysis of *APRT* diploid genotypes (Fig. 5d). Although TIDE analysis revealed compound heterozygous genotypes including indel and NHEJ alleles (as seen in Fig. 4e), we focused only on biallelic editing, or monoallelic editing where the alternate allele was normal. Monoallelic editing was biased in clones from mCh^low^ sorting (Fig. 5d, top), while biallelically edited clones were more prevalent from mCh^high^ (Fig. 5d, bottom). Thus, the simultaneous isolation of an allelic series in iPSC which have been handled under equivalent experimental conditions provides a new source of monoallelic (APRT*J/Norm and Silent/Norm) and biallelic (APRT*J/Silent and APRT*J/APRT*J) isogenic parity (Fig. 5e).

**Table 4:** APRT allele spectrum of excised populations following FACS enrichment

		Non-targeted		NHEJ	MMEJ		
mCh Pop.	Samples analyzed	Normal allele	Indel	NHEJ (perfect)	Silent ONLY	APRT*J & Silent	Fidelity (%)
low	56	1	21	25 (10)	2	7	16.1
high	49	2	3	34 (21)	1	9	20.4

## Discussion

Microhomology-mediated end joining reproducibly deletes one copy of tandem homology along with intervening genomic DNA sequences to generate deletions of predictable size^9,15,31^. In the current study, we report the development of a scarless genome editing approach termed MhAX (microhomology assisted excision), where artificially engineered µH accompanied by nested CRISPR-Cas9 target sites predisposes DSBR toward scarless excision of a selectable marker. Demonstrations of monoallelic and biallelic editing to deposit disease-relevant HPRT_Munich_ or APRT*J mutations highlight the precision of this endogenous pathway. Based on conventional donor construction and standard gene targeting principles which have been employed in the field for decades^36,37^, MhAX provides a tractable methodology which enhances established gene targeting pipelines. When recruiting HDR to deposit point mutations, dsDNA donors present an advantage over ssDNA through extension of the conversion tract from tens to hundreds of bases from the DSB^38^. Our approach is complementary to NHEJ or MMEJ mediated insertion of transgenic cassettes flanked with minimal homology arms following nuclease cleavage of both the donor and target genome^39–41^. Analogous to recombinase-based cassette removal techniques, yet completely independent of residual exogenous recombinase sites^42^, MMEJ-based transgene excision could have similar broad applications in the precise elimination of foreign genetic elements for gene or cell therapy applications, and possibly even conditional gene manipulation.

In this initial demonstration of MhAX, we achieved excision rates from ~5–35%, which is practical for clonal isolation of iPSCs with biallelic modification. In human iPSCs, we empirically verified published observations that longer µH improves MMEJ repair rates^43^. NHEJ deletions ranging from 0.5–8 kb of the MALAT1 gene using CRISPR-Cas9 and paired sgRNAs in human H9 ESCs showed an inverse correlation between deletion size and efficiency^44^, suggesting that consolidation of selection markers to reduce cassette size (here ~5 kb) may further improve excision rates. Additionally, µH characteristics such as GC-content or reduced distance from the break site (heterology)^45,46^ may affect MMEJ. Heterologous tails of 8–9 bp were shown to be less inhibitory to MMEJ in mammalian cells than yeast^47^, and our data indicates that heterology >7 bp impedes, but does not completely prevent MMEJ in human iPSCs. Heterology could be theoretically reduced to zero by overlapping, rather than abutting, endogenous and operational sequences. It should be noted that the extent to which these parameters warrant manipulation may depend upon the sequence context of the target locus, and that for additional interrogation of DSBR processes in human iPSCs, our HPRT reconstitution assay could prove effective.

MhAX uses unique CRISPR-Cas9 protospacers and cognate sgRNAs for excision (here ps1, targeting eGFP-derived sequences), which may be further optimized for high activity, low cytotoxicity, and reduced homology to the host genetic background, using parameters defined from large scale screens^25^. A comparable ssODN-based scarless editing method, which requires two rounds of targeting to generate point mutations, demands the design and assay of specialized CRISPR-Cas9 sgRNAs which are limited by the target locus^48^. Moreover, those sgRNAs retain high similarity to edited alleles and were shown to re-cleave them at low frequency. Custom MhAX sgRNAs, on the other hand, can be freely designed to have consistent cleavage activity and restricted off-target profiles. Moreover, since protospacers employed in MhAX are completely removed from the genome after excision, both the corrected and mutant alleles are protected from subsequent cleavage events, allowing the sgRNAs to be recycled after their initial use. Further improvements in protospacer prediction^49^ and CRISPR-Cas9 engineering^50^ will continue to aid reagent design.

Gene targeting may be streamlined using fluorescent enrichment for HDR and against random donor plasmid integration^34^. In the current work, we combined gene-trap selection with constitutive expression of CAG::GFP to exclude random integration, and CAG::mCherry in order to track targeting and excision in populations, without the need for intermediate cloning. Furthermore, separation of the bimodal mCh^pos^ population ultimately enriched for monoallelically and biallelically edited iPSCs. In cases where consistent scaling of reporter gene expression between heterozygous and homozygous targeting may not be observed, isolation of biallelically modified iPSC clones could be achieved using dual-fluorescent, or dual-drug positive selection^16^. FACS for mCh^neg^ iPSCs, along with PCR and more conclusive Southern blot genotyping provided evidence that excised transgenes do not readily re-insert into the genome, presenting a potential advantage over transposition^51,52^, which also requires retention of a proximal transposon footprint.

Genetic background has been implicated in contributing the greatest source of variation between iPSC lines^53,54^, such that debate over what constitutes appropriate controls remains. The creation of isogenic controls directly from patient or normal iPSCs constrains genetic backgrounds facilitated by genome engineering^3.^ However experimental manipulations such as nuclease exposure, extensive subcloning, and prolonged passage may additionally contribute to subtle deviations from the original parental cell line^10^. Using a plasmid recircularization assay, mismatched base conversion within MMEJ junctions has been shown to occur in yeast^32^, reminiscent of our observations at the human *HPRT1* locus. Our intentional use of imperfect µH to direct MhAX allowed the isolation of both mutant and normal isogenic clones from a single experiment. Thus, gene editing using the MhAX technique combined with unilaterally-engineered point mutations retains the closest possible relation between two clonal cell lines, opening a new dimension to the interdependence of isogenic controls.

## Methods

### Plasmid construction

Supplementary Table 3 provides a list of sequence-verified plasmids used in this study. Primers used for cloning and validation are listed in Supplementary Tables 4-6. Complete sequences are available through Addgene or upon request. Detailed cloning histories are available upon request as Snapgene files.

*HPRT1*_B NC-TALENs were described previously^13^. Avr-TALEN expression vectors with non-repeat-variable di-residue (non-RVD) variations were assembled using the Platinum TALEN method^18^, into a modified ptCMV-136/63-VR expression vector containing a CAG promoter instead of CMV. The DNA-binding modules were then assembled using the two-step Golden Gate method. Assembled modules were as follows: Left, HD HD NI NG NG HD HD NG NI NG NN NI HD NG NN NG NI NN NI NG; Right, NI NG NI HD NG HD NI HD NI HD NI NI NG NI NN HD NG.

For CRISPR-Cas9 expression, sgRNA oligos were annealed and cloned into pX330 (Addgene plasmid #42230, a gift from Feng Zhang) linearized with *Bbs*I as previously described^22^. The resulting plasmids were sequence verified using primer dna790.

The *HPRT1* SSA reporter vector was used as previously described^13^. Additional SSA reporter vectors for AAVS1 TALENs and eGFP sgRNAs were generated by annealing oligos consisting of the target genomic sequence followed by ligation into pGL4-SSA linearized with *Bsa*I.

To derive homology for *HPRT1* gene editing, a larger region of 1253 bp surrounding the *HPRT1*_B TALEN target site was PCR amplified from 201B7 iPSC genomic DNA^55^, cloned into a minimal pBluescript backbone, and sequence verified. To derive homology for *APRT* gene editing, a larger region of 1256 bp was PCR amplified from 1383D6 iPSC genomic DNA, cloned using the Zero Blunt TOPO PCR Cloning Kit for Sequencing (Invitrogen), and sequence verified. The resulting plasmid (pCR4-hAPRT-G) represents the rs8191489 G allele.

The puroΔTK selection marker was designed as previously described^56^, and constructed in an AAVS1 donor vector (Addgene plasmid #22075, a gift from Rudolf Jaenisch). A modified version (KW999) containing the CAG::mCherry reporter and unique flanking restriction sites was constructed using pAAVS1-P-CAG-mCh (Addgene plasmid #80492) as a base. The pCAG-eGFP-pA plasmid (KW991) used as a negative-selection backbone was constructed by Gateway cloning (Invitrogen) of a pENTR-eGFP Entry vector.

Two different strategies employing one-pot InFusion cloning (Clontech) were used to generate *HPRT1* and *APRT* donor vectors. For plasmids KW836, KW838, and KW883 the p3-HPRT1 vector was inverse-PCR amplified with primers that included all operational sequences for excision and MMEJ repair, including: the ps1 protospacer and PAM sequences, appropriately engineered µH, as well as Silent and Munich mutations, and terminating with 12–15 nt InFusion overhangs. For KW794, a version of the p3-HPRT1 homology plasmid containing an MC1::DTA negative selection marker was used as a template. The 2A-puroΔTK selection marker was amplified such that the T2A and selection marker coding region were in-frame with *HPRT1* exon 3. All PCR-amplified regions were verified by sequencing. The CAG::mCherry reporter was introduced directly by restriction-ligation or sequentially through a CAG::Gateway cassette from pAAVS1-P-CAG-DEST (Addgene plasmid #80490), followed by Gateway cloning of mCherry. For plasmids KW883, KW1005, KW1033, and KW1034, left and right homology arms were PCR amplified to include InFusion homologies, ps1 protospacer and PAM sequences, and appropriately engineered µH. The plasmid backbone and selection/reporter cassette were prepared as restriction fragments from KW991 (*Acc*65I) and KW999 (*Bgl*II + *Hind*III), respectively. The four fragments were assembled in a single InFusion cloning reaction, and PCR-amplified regions were verified by sequencing.

MMEJ assay plasmids were constructed by InFusion cloning. The Ampicillin selection marker was initially swapped for Kanamycin to generate pGL4K. For µH sizes of 0—30 bp, the ps1 protospacers and PAM sequences were introduced with the µH by inverse PCR of the pGL4K plasmid, and the Cam^R^/ccdB selection marker was amplified with common primers. For µH sizes of 40 and 50 bp, part of the µH was added to the Cam^R^/ccdB amplicon.

### SSA and MMEJ assays

HEK293T cells (Thermo Scientific) were maintained in culture medium containing DMEM, 10% FBS, penicillin-streptomycin, and L-glutamine. SSA assays were carried out as previously described^19^, and MMEJ assays followed a similar design. Briefly, DNA mixtures containing 200 ng each of TALEN or CRISPR-Cas9 nuclease expression vectors, 100 ng of the appropriate pGL4-SSA (SSA assay) or pGL4K-MMEJ-eGFP1-µX (MMEJ assay) target vector, and 20 ng pGL4_74_hRlucTK Renilla reference vector (Promega) were prepared in 25 µL of Opti-MEM I reduced-serum medium (Invitrogen) in a 96-well plate. Twenty five microliter of Opti-MEM I containing 0.7 µL of Lipofectamine 2000 (Invitrogen) was then added, and incubated at room temperature for 15 min. HEK293T cells were then added at a density of 4 × 10^4^ cells per 100 µL in culture medium, and incubated at 37 °C, 5% CO_2_ for 24 h. To assay luciferase activity, plates were first equilibrated to room temperature before replacing 75 µL of growth medium with 75 µL of Dual-Glo reagent (Promega). After 10 min incubation, 150 µL of reaction was transferred to a white microtitre plate, and luminescence (1 s) was read on a Centro LB960 (Berthold) or 2104 EnVision Multilabel Plate Reader (Perkin Elmer). Following the addition of 75 µL Stop reagent and 10 min incubation, Renilla luminescence was similarly read. Relative luminescence was calculated by the ratio of Firefly/Renilla intensity.

### T7EI assay

T7EI assays were performed as previously described^57^ with some modifications. APRT nuclease expression plasmids (1 µg each) and 6 µL FugeneHD (Promega) were incubated in 150 µL of Opti-MEM I at room temperature for 15 min. HEK293T cells (3 × 10^5^ in 300 µL Opti-MEM I) were incubated with the transfection complexes at room temperature for 5 min before plating in a 6-well dish in culture medium. After incubation at 37 °C, 5% CO_2_ for 96 h, cells were harvested and genomic DNA was isolated using the DNeasy Blood & Tissue Kit (Qiagen) according to the manufacturer’s instructions. The *APRT* locus was amplified from 100 ng of genomic DNA by PCR with Phusion Hot Start II High-Fidelity DNA Polymerase (Thermo Scientific) using the primers listed in Supplementary Table 7. The resulting PCR products were purified by Agencourt AMPure XP (Beckman Coulter) according to the manufacturer’s instructions. 200 ng purified PCR products were denatured in NEBuffer 2 (New England Biolabs) at 95 °C for 5 min and re-annealed at a controlled rate of −0.1 °C/s. Samples were divided in half and 1 µL of T7 Endonuclease I (New England Biolabs) or ddH_2_O was added followed by incubation at 37 °C for 15 min. The T7EI reaction was stopped with 0.25 M EDTA. Digestion products were analyzed by gel electrophoresis and indel frequencies were calculated by densitometry in ImageJ (NIH) using the formula: 100 × (1—(1—(*b* +* c*) / (*a* + *b* + *c*))^1/2^).

### Human ESC and iPSC culture

Undifferentiated human ESCs and iPSCs were maintained under feeder-free conditions as described previously^58^. Briefly, H1 human ESCs^17^ and 1383D6 human iPSCs^16^ were cultured on recombinant human Lamin-511 E8 fragment (iMatrix-511, Nippi) coated 6-well tissue culture dishes (0.5 μg/cm^2^) in StemFit AK03 or AK02N (AJINOMOTO) medium. For passaging, cells were detached by treatment with 300 μL Accumax (Innovative Cell Technologies, Inc.) at 37 °C for 10 min, followed by gentle mechanical dissociation with a pipette. To collect the cells, 700 μL of culture medium containing 10 μM ROCK inhibitor, Y-27632 (Wako) was added. Cells were counted using trypan blue exclusion on a TC20 (Bio-Rad). Typically, 1–3 × 10^3^ cells per cm^2^ were seeded on each passage in media containing Y-27632. After 48 h culture, the medium was changed without Y-27632. Five to seven days after plating, the cells reached 80–90% confluency and were again prepared for passage. To make frozen stocks, iPSCs were resuspended at a density of 1 × 10^6^ viable cells per 1 mL STEM-CELLBANKER (Takara) and 200—500 μL of cell suspension (2–5 × 10^5^ iPSC) was transferred to a cryogenic tube. Stock vials were defrosted onto iMatrix-511 coated 6-well tissue culture dishes (one vial per 10 cm^2^) in StemFit AK03 or AK02N medium containing Y-27632.

Maintenance of 409B2 human iPSCs^59^ was carried out on SNL feeder cells^60^ in Primate ES Cell medium (ReproCELL). For passaging, SNL feeder cells were detached from the well by incubation with 300 μL CTK solution containing 1 mg/mL collagenase, 0.25% trypsin, 20% KSR, and 1 mM CaCl2 in Dulbecco’s phosphate buffered saline (DPBS), Mg^2+^ and Ca^2+^ free (Nacalai Tesque) for 2 min at room temperature. CTK solution was then removed and wells were washed twice with 2 mL DPBS. 1 mL of Primate ES Cell medium supplemented with Recombinant Human FGF-basic (PEPROTECH) was added and colonies were collected with a cell scraper and dissociated into small clumps by pipetting up and down a few times throughout the entire well. The split ratio was ~1:5 to a fresh SNL feeder-coated dish.

### HPRT disruption with TALENs

*HPRT1* knockout experiments using NC-TALENs in 409B2 iPSCs were carried out on SNL feeders with delivery of DNA by Neon (Invitrogen) electroporation as previously described^13^. TALEN evaluation assays and *HPRT1* knockout experiments using Avr-TALEN in H1 ESCs and 1383D6 iPSCs were carried out under feeder-free conditions with delivery of DNA by NEPA21 (Nepa Gene Co., Ltd) as previously described^16^. Briefly, CAG-dNC-HPRT1 TALENs (3 µg each) or CAG-Avr-HPRT TALENs (3 µg each) were transfected by NEPA21 electroporation into 1 × 10^6^ cells in a single-cell suspension. Electroporated cells were plated at a density of 1–5 × 10^5^ cells / 60 mm culture dish. Two days after electroporation, 6-thioguanine (6-TG, 20 µM; Sigma-Aldrich) selection was initiated, with daily feeding over a period of 7–10 days. For population analyses, at cultures of at least 50–300 colonies were pooled for genomic DNA preparation. For clonal analyses, iPSC colonies were picked in 5-10 µL AK02N medium using a micropipette and transferred with gentle dissociation into a 96-well tissue culture plate containing AK02N medium (10 µM Y-27632). Isolated clones were incubated at 37 ^o^C, 5% CO_2_ for 6-10 days in AK02N medium. At 80-90% confluency, the well was washed with 100 µL DPBS and incubated with 30 µL of Accumax for 20-30 min at 37 ^o^C, 5% CO_2_. Accumax was removed and colonies were dissociated with a multichannel micropipette in 100 µL of AK02N medium containing 10 µM Y-27632. Cell suspensions were split evenly for FACS analysis, gDNA isolation, or freezing in foil-wrapped 96-well plates in STEM-CELLBANKER (TaKaRa) at -80 ^o^C previously described^58^. Selected clones were defrosted and expanded for permanent storage in liquid nitrogen.

### iPSC gene targeting

Gene targeting was carried out essentially as described^16^. Briefly, nuclease expression vectors (1 µg each TALEN or Cas9/sgRNA plasmid) and donor vectors (3 µg) were transfected by NEPA21 electroporation into 1 × 10^6^ cells in single-cell suspension. Electroporated iPSCs were plated at a density of 1–5 × 10^5^ cells per 60 mm culture dish in Stemfit media containing Y-27632. Two days after electroporation, Y-27632 was removed and 0.5 µg/mL puromycin (Sigma-Aldrich) added, with daily feeding over a period of 7–10 days. Clones were isolated manually with a micropipette and processed under feeder-free conditions in 96-well format as described above. For FACS-based MhAX gene editing, puro^R^ colonies were pooled and passaged once in bulk or directly subjected to FACS. Genotyping by 5′, 3′ and spanning genomic PCR, junction sequencing, and Southern blot is described below.

### Cassette excision

To initiate cassette excision, 1 or 3 µg of pX-ps1 expression vector was transfected by NEPA21 electroporation into 1 × 10^6^ cells in single-cell suspension, and plated at a density of 1–5 × 10^5^ cells per 60 mm culture dish in Stemfit media containing Y-27632. Two days after electroporation, Y-27632 was removed.

Cassette excision enriched by HAT selection (1 × ) (Sigma-Aldrich or Gibco) was carried out with daily feeding over a period of 7–10 days. Selection with FIAU against the ∆TK marker in the 2A-puro∆TK gene-trap cassette was inefficient, and therefore not used to enrich for excised clones in this study. Enrichment of cassette-excised mCh^neg^ cells by FACS was performed. iPSCs electroporated with pX-ps1 were plated as usual and allowed to recover in the absence of selective pressure. After 5 to 7 days, cells were subjected to FACS sorting as described below. Recovered mCh^neg^ cell populations were counted and plated for bulk analysis or at clonal density (400–800 cells per 60 mm dish) in the absence of HAT (1×). iPSCs under HAT selection were plated at a 10-fold higher density (4000–8000 cells per 60 mm dish) than unselected in order to obtain similar colony numbers. Clones were isolated manually and processed under feeder-free conditions in 96-well format as described above. Genotyping by PCR, sequencing, and Southern blot is described below.

### Flow cytometry and cell sorting

For routine measurement of GFP or mCherry fluorescence intensities, ~3.0 × 10^5^ cells were suspended in FACS Buffer (DPBS supplemented with 2% FBS) and analyzed using a BD LSRFortessa Cell Analyzer (BD Biosciences) with BD FACSDiva software (BD Biosciences). mCherry fluorescence intensities of targeted clones were measured in 96-well format on a MACSQuant VYB (Miltenyi Biotec).

For the isolation of targeted GFP^neg^ / mCh^pos^ targeted, or cassette-excised mCh^neg^ iPSCs, cells were harvested as a single-cell suspension in FACS Buffer at a density of ~1 × 10^6^ cells per mL and filtered through a cell-strainer to remove clumps. After setting gates for singlets, the desired population was collected on a BD FACSAria II cell sorter (BD Biosciences) into Stemfit AK02N medium containing 10–20 µM Y-27632. Sorting efficiencies were determined using a BD LSRFortessa Cell Analyzer.

Flow cytometry data were analyzed and generated by FlowJo software v9.7.6 or higher (Tree Star).

### Genomic DNA isolation

Genomic DNA from populations or clones for PCR screening and sequencing was extracted from 0.5—1 × 10^6^ cells using a DNeasy Blood & Tissue Kit (Qiagen) according to the manufacturer’s instructions. Genomic DNA for Southern blotting of clones was extracted from one confluent well of a 6-well dish (at least 3 × 10^6^ cells) using lysis buffer (100 mM Tris-HCl, pH 8.5, 5 mM EDTA, 0.2% SDS, 200 mM NaCl, and 1 mg/mL Proteinase K), followed by standard phenol/chloroform extraction, ethanol precipitation, and resuspension in TE pH 8.0. For high-throughput Southern blotting or PCR screening, genomic DNA was extracted in 96-well format^61^ using plate lysis buffer (10 mM Tris-HCl, pH 7.5, 10 mM EDTA, 0.5% sarcosyl, 10 mM NaCl, and 1 mg/mL Proteinase K) followed by direct ethanol precipitation and re-suspension in restriction digestion mix or TE pH 8.0. For clonal analyses from mCh^low^ and mCh^high^ FACS-sorted populations, each iPSC colony was manually picked with a micropipette in a 5 µL volume of media and added to 10 µL of QuickExtract DNA Extraction Solution (Epicenter). Samples were processed by heating to 65 °C for 6 min then 98 °C for 2 min prior to PCR.

### PCR genotyping

Genomic PCR was performed using AmpliTaq 360 (Applied Biosystems), KAPA HiFi, or KAPA Taq Extra (KAPA Biosystems) on a Veriti 96-well Thermal Cycler (Applied Biosystems) or Biometra Trio (Analytik Jena) according to the manufacturer’s instructions. Specific PCR conditions are available upon request.

Primers used to verify gene targeting events are described in Figs. 2 and 4 and Supplementary Figs. 10 and 11, and Supplementary Table 7 and 8. Sequencing of the junction regions was used to ensure the fidelity of the flanking µH and CRISPR-Cas9 protospacers. PCR products from cassette-excised iPSC clones were sequenced directly, while PCR products from populations were cloned using the Zero Blunt TOPO PCR Cloning Kit for Sequencing (Invitrogen), and then amplified and sequenced from the resulting bacterial colonies with Ex Taq DNA Polymerase (Takara) and T3/T7 primers. Primer design for exons 1–9 of *HPRT1* (Accession NG_012329.1) and exons 1-5 of *APRT* (Accession NG_008013.1) was performed using the NCBI Primer-BLAST with optional settings for human repeat filter, SNP handling, and primer pair specificity checking to *H.sapiens* (taxid:9606) reference genome, and are listed in Supplementary Table 9.

### Sequencing

PCR products were treated with ExoSAP-IT (Affymetrix) prior to sequencing. DNA sequencing was performed using BigDye Terminator v3.1 Cycle Sequencing Kit (Applied Biosystems), purification by ethanol precipitation, and run on a 3130*xl* Genetic Analyzer (Applied Biosystems). Sequence alignments were performed using Sequencher v5.1 (Genecodes) or Snapgene v3.1.4 or higher (GSL Biotech LLC.). Sequence trace files with poor base calling confidence were excluded from further analyses.

### TIDE analysis

TIDE analysis of mixed sequences was performed using the online tool at https://tide.nki.nl/^20^, or as a stand-alone R Script. Sequence data from H1 ESCs or 1383D6 iPSCs was used as a reference. For *HPRT1* indels, populations of ESCs or iPSCs consisting of ~50 clones (H1) or 200 clones (1383D6) were pooled and harvested for genomic DNA and amplified as described above. As TIDE is designed for CRISPR-Cas9, whereas TALENs induce DSBs at an undetermined position within the spacer, we positioned the predicted breakpoint at the 5′ end of the spacer adjacent the *HPRT1*_B TALEN-L binding site (TTCCTATGACTGTAGAT^TTT) where base-calling confidence initially dropped co-incident with visibly mixed sequence. The deletion size window was extended to 20 bp to accommodate larger deletions. For resolving heterozygous *APRT* clones, the APRT sgRNA-2 sequence was used to set the breakpoint and the deletion window was extended to 50 bp in order to accommodate predicted NHEJ events. The remaining parameters were set to default or allowed to adjust automatically based on the properties of the sequence trace files provided.

### RFLP analyses

In order to verify deposition of Silent mutations following excision with unilaterally or bilaterally mutant µH, genomic DNA was amplified using primer set dna1720/411 (*HPRT1*) or dna1711/1712 (*APRT*). Cleaved amplicons were resolved by gel electrophoresis following treatment with or without *Afl*II (*HPRT1*) or *Acc*65I (*APRT*) restriction enzyme (Fermentas).

### Southern blotting

The HPRT-B and APRT-5′ genomic, and mCherry transgenic probe fragments were prepared from genomic or plasmid PCR amplicons, respectively (Supplementary Table 10). DIG labeled dUTP (Roche) was incorporated by PCR amplification using Ex Taq (Takara) according to the manufacturer’s instructions.

Genomic DNA (5–10 µg) was digested with 3-fold to 5-fold excess restriction endonuclease (Fermentas) overnight in the presence of BSA (100 µg/mL), RNaseA (100 µg/mL) and spermidine (1 mM). Digested DNA fragments were separated on a 0.8% agarose gel, depurinated, denatured, and transferred to a Hybond N + nylon membrane (GE Healthcare) using 20 × SSC. The membrane was UV crosslinked, pre-hybridized, and incubated with 150 ng digoxigenin (DIG)-labeled DNA probe in 4 mL DIG Easy Hyb buffer (Roche) at 42 °C overnight with constant rotation. After repeated washing at 65 °C (0.5 × SSC; 0.1% SDS), the membrane was blocked (DIG Wash and Block Buffer Set, Roche) and alkaline phosphatase-conjugated anti-DIG antibody (1:10,000, Roche) was applied to a membrane. Signals were raised by CDP-star (Roche) and detected by ImageQuant LAS 4000 imaging system (GE Healthcare). Uncropped images for Southern blot data presented in Figs. 2 and 4 are shown in Supplementary Fig. 19.

### Microscopy

Phase-contrast and fluorescence images were acquired on a BZ-X710 (KEYENCE) using appropriate filters and exposure times.

### Drug sensitivity assays

iPSC lines edited at the *HPRT1* locus and controls were plated at 3 × 10^4^ cells per well in a 6-well culture dish, grown for 2 days without HAT, followed by 2 additional days with or without HAT. Cells were harvested on days 2, 3, and 4 post-plating, and re-suspended in 100 µL of AK02N. An 11 μL aliquot of cell suspension was mixed 1:1 with Trypan Blue Stain 0.4% (Gibco) by gentle pipetting, and 10 μL was applied to each side of a Counting Slide (Bio-Rad). Cell numbers were determined with the TC20 Automated Cell Counter (Bio-Rad).

iPSC lines edited at the *APRT* locus and controls were plated at 1 × 10^5^ cells per well in a 6-well culture dish in media containing Y-27632 and grown for 2 days. 2, 6-diaminopurine (DAP; Sigma) dissolved in DMSO was added to growth media at a final concentration of 10 µg/mL for an additional 2 days, after which the wells were stained with crystal violet and imaged. Treatment with DMSO was used as a control.

### Crystal violet staining

Confluent or drug-selected iPSC culture dishes were washed twice with ice-cold DPBS and fixed by ice-cold methanol (Nacalai Tesque) for 10 min at room temperature. The methanol was removed and sufficient crystal violet solution (HT90132, Sigma-Aldrich) was added to cover the bottom of the dish. After 10 min incubation at room temperature, the staining solution was removed and the dishes were gently rinsed with water. After complete drying at room temperature, whole well images were acquired with a STYLUS XZ-2 (OLYMPUS) camera.

### Western blotting

For HPRT protein analysis, total cell lysates were prepared by heating 1 × 10^6^ cells to 70 ^o^C for 10 min in 100 µL NuPAGE LDS Sample Buffer (1×) (Thermo Fisher Scientific) containing DTT at a final concentration of 50 mM. Lysates were resolved on Bis-Tris gels, and probed using HPRT (F-1, sc-376938, 1:200, Santa Cruz) and anti-actin (A2066, 1:5,000, Sigma Aldrich) antibodies. Sheep anti-Mouse IgG, HRP-Linked Whole Ab (GE Life Science:NA931-100UL) and goat anti-rabbit IgG-HRP (Santa Cruz: sc-2004) secondary antibodies for HPRT and anti-actin, respectively, were used at 1:5000 dilution. Signals were raised using ECL Prime Western Blotting Detection Reagent (GE Healthcare) and detected on an ImageQuant LAS 4000 imaging system (GE Healthcare).

### Metabolome analysis

The concentrations of all the charged metabolites in media samples were measured by capillary electrophoresis time-of-flight mass spectrometry (CE-TOFMS, Agilent Technologies, Santa Clara, CA) using the methods developed by the authors^29,30^. For media sample preparation, 1.5 × 10^5^ cells from the indicated iPSC clones were seeded in 150 µL of AK02N medium containing Y-27632 (10 µM) per well of a 96-well plate and cultured at 37 °C, 5% CO_2_. The next day, the medium was replaced with 150 µL of fresh AK02N medium without Y-27632. Media-only reference samples were prepared and similarly incubated at 37 °C, 5% CO_2_. After 24 h, 100 µL of spent medium was collected and mixed with 400 μL of methanol containing L-methionine sulfone (Wako), MES (Dojindo), and CSA (Wako) internal standards (200 μM each). Following the addition of 200 μL Milli-Q ultrapure water, the samples were extracted with 500 μL chloroform. The aqueous layer was subjected to 5 kDa ultrafiltration (HMT) and lyophilized (LABCONCO). Lyophilized samples were resuspended in 50 μL Milli-Q ultrapure water containing 3-Aminopyrrolidine (Sigma-Aldrich) and Trimesate (Wako) internal standards (200 μM each) before analysis.

To analyze cationic compounds, a fused silica capillary (50 μm i.d. × 98 cm) was used with 1 M formic acid as the electrolyte^62^. Methanol / water (50% v/v) containing 0.01 μM hexakis (2,2-difluoroethoxy) phosphazene was delivered as the sheath liquid at 10 μl/min. ESI-TOFMS was performed in positive ion mode, and the capillary voltage was set at 4 kV. Automatic recalibration of each acquired spectrum was achieved using the masses of the reference standards ([13C isotopic ion of a protonated methanol dimer (2MeOH+H)]+, *m*/*z* 66.0631) and ([hexakis (2,2-difluoroethoxy) phosphazene+H]+, *m*/*z* 622.0290). To identify metabolites, relative migration times of all peaks were calculated by normalization to the reference compound, 3-aminopyrrolidine. The metabolites were identified by comparing their *m*/*z* values and relative migration times to the metabolite standards. Quantification was performed by comparing their peak areas to calibration curves generated using internal standardization techniques with methionine sulfone. The other conditions were identical to those described previously^29^.

To analyze anionic metabolites, a commercially available COSMO(+) (chemically coated with cationic polymer) capillary (50 μm i.d. × 105 cm) (Nacalai Tesque, Kyoto, Japan) was used with a 50 mM ammonium acetate solution (pH 8.5) as the electrolyte. Methanol/5 mM ammonium acetate (50% v/v) containing 0.01 μM hexakis (2,2-difluoroethoxy) phosphazene was delivered as the sheath liquid at 10 μL/min. ESI-TOFMS was performed in negative ion mode, and the capillary voltage was set at 3.5 kV. For anion analysis, trimesate and D-Camphor-10-sulfonic acid (CAS) were used as the reference and the internal standards, respectively. The other conditions were identical to those described previously^30^.

The data were analyzed and quantified using in-house software (Master Hands-2.17.1.11) developed particularly for CE-TOFMS-based metabolomic data analysis.

### Data availability

Previously published data used in primer design for exons 1-9 of *HPRT1* and exons 1-5 of *APRT* are available from NCBI Genbank under accession codes NG_012329.1 and NG_008013.1, respectively.

Plasmids used in this study are available through Addgene (107271 - 107281) or upon request. Complete CE-TOFMS metabolomics data and DNA sequence alignments are available upon request. All other data are available upon request.

## Electronic supplementary material

Supplementary Information
