# Supplementary material for: Microhomology-assisted scarless genome editing in human iPSCs
Source: Nat Commun. 2018 Mar 5;9:939. doi: 10.1038/s41467-018-03044-y (PMC5838097; doi:10.1038/s41467-018-03044-y)
Supplement: Supplementary file 1 — Supplementary Information [file 41467_2018_3044_MOESM1_ESM.pdf]

SUPPLEMENTARY INFORMATION

Microhomology-assisted scarless genome editing in human iPSCs

Shin-Il Kim, Tomoko Matsumoto, Harunobu Kagawa, Michiko Nakamura, Ryoko Hirohata, Ayano Ueno, Maki Ohishi, Tetsushi Sakuma, Tomoyoshi Soga, Takashi Yamamoto, and Knut Woltjen

This file includes:

Supplementary Figures 1-19

Supplementary Tables 1-10

Supplementary References

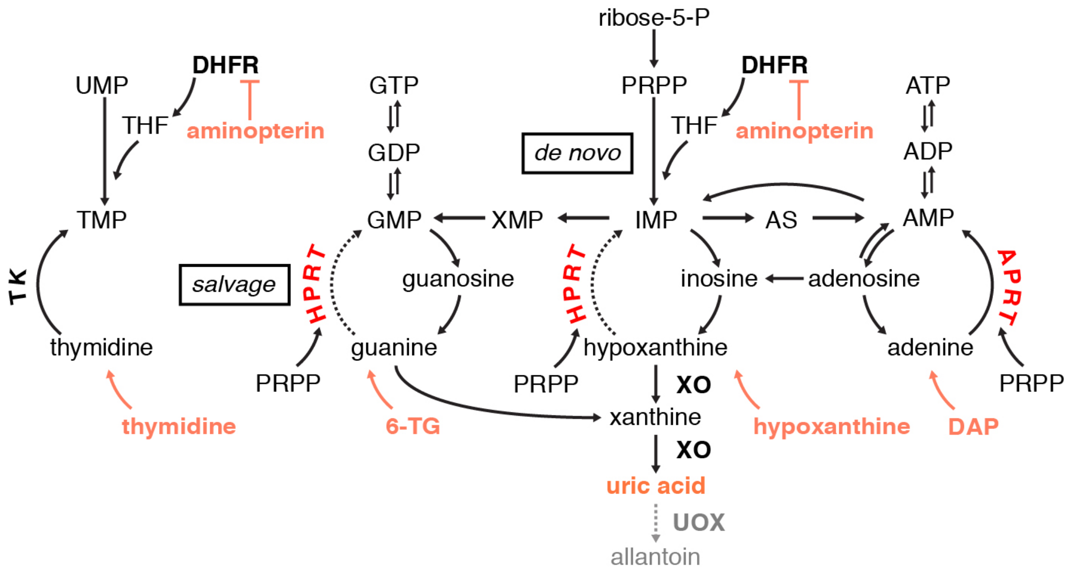

Supplementary Figure 1. Purine biosynthesis pathways and metabolic selection.

De novo synthesis and salvage pathways in purine metabolism. Hypoxanthine phosphorybosyltransferase (HPRT) catalyzes both the conversion of guanine to guanine monophosphate (GMP), and hypoxanthine to inosine monophosphate (IMP). With complete or partial HPRT deficiency, guanine and hypoxanthine metabolites are expected to accumulate. Xanthine oxidase (XO) converts hypoxanthine into uric acid. Unlike most mammals, humans lack uric acid oxidase (UOX) and do not enzymatically convert uric acid into allantoin, leading to hyperuricemia. Adenine phosphoybosyltransferase (APRT) catalyzes the conversion of adenine to adenine monophosphate (AMP), and prevents accumulation of 2,8-dihydroxyadenine (2,8-DHA). At high concentrations 2,8-DHA forms crystals resulting in kidney stones, and in severe cases can cause kidney failure and urolithiasis.

Metabolic selection for HPRT activity and inactivity is carried out using media containing hypoxanthine, aminopterin, and thymidine (HAT), or 6-thioguanine (6-TG), respectively. Blocking dihydrofolate reductase (DHFR) activity with aminopterin prevents *de novo* synthesis and forces cells to rely wholly on hypoxanthine salvage by HPRT. On the other hand, active HPRT incorporates 6-TG into DNA synthesis and cell signaling pathways, leading to cytotoxicity. Metabolic selection for APRT inactivity is carried out using 2’6’-diaminopurine (DAP), a purine analogue toxic to cells competent for adenine salvage. As APRT is solely responsible for salvage of adenine, counter-selection for its activity is possible by blocking de novo synthesis of IMP with azaserine, or the conversion of IMP to AMP with alanosine.

Additional abbreviations: ADP, ATP, adenine di-, triphosphate; GDP, GTP, guanine di-, triphosphate; PRPP, 5-Phospho-D-ribose 1-diphosphate; THF, tetrahydrofolate; TMP, thymidine monophosphate; UMP, uracil monophosphate.

Figure adapted from http://www.lesch-nyhan.org/en/definition/biochemistry/hprt with permission from J.E. Visser, MD, PhD and H.A. Jinnah, MD, PhD.

```
DELETIONS
409B2      aaatagtgatagatCCATTCCTATGACTGTAGATtttatcagactgaagAGCTATTGTGTGAGT-ATatttaatatatgat         REF
409-LR2a   aaatagtgatagatCCATTCCTATGACTGTAGATtttatc-gactgaagAGCTATTGTGTGAGT-ATatttaatatatgat         (-1)
409-LR2b   aaatagtgatagatCCATTCCTATGACTGTAGATtttatca--ctgaagAGCTATTGTGTGAGT-ATatttaatatatgat         (-2)
409-LR3a   aaatagtgatagatCCATTCCTATGACT-----------------gaagAGCTATTGTGTGAGT-ATatttaatatatgat         (-17)
409-LR3b   aaatagtgatagatCCATTCCTATGACTGTAGATttta---gactgaagAGCTATTGTGTGAGT-ATatttaatatatgat         (-3)
409-LR4a   aaatagtgatagatCCATTCCTATGACTGTAGATtttat------gaagAGCTATTGTGTGAGT-ATatttaatatatgat         (-6)
409-LR4b   aaatagtgatagatCCATTCCTATGACT-----------------gaagAGCTATTGTGTGAGT-ATatttaatatatgat         (-17)
409-LR5b   aaatagtgatagatCCATTCCTATGACTGTAGATtttatc-gactgaagAGCTATTGTGTGAGT-ATatttaatatatgat         (-1)
409-LR6a   aaatagtgatagatCCATTCCTATGACTGTAGATtttatc----tgaagAGCTATTGTGTGAGT-ATatttaatatatgat         (-4)
409-LR6b   aaatagtgatagatCCATTCCTATGACTGTAGATtttatc----tgagaAGCTATTGTGTGAGT-ATatttaatatatgat         (-4)
409-LR10a  aaatagtgatagatCCATTCCTATGACTGTAGATtttatc----tgaagAGCTATTGTGTGAGT-ATatttaatatatgat         (-4)
409-LR10b  aaatagtgatagatCCATTCCTATGACTGTAGATttta----actgaagAGCTATTGTGTGAGT-ATatttaatatatgat         (-4)
409-LR11a  aaatagtgatagatCCATTCCTATGACTGTAGATtttatcagactgaagAGCTATTGTGTGAGT-ATatttaatatatgat         NORM
409-LR11b  aaatagtgatagatCCATTCCTATGACTGTAGATt---------------------GTGTGAGT-ATatttaatatatgat         (-21)
409-LR12a  aaatagtgatagatCCATTCCTATGACTGTAGATtttatca--ctgaagAGCTATTGTGTGAGT-ATatttaatatatgat         (-2)
409-LR12b  aaatagtgatagatCCATTCCTATGACTGTAGA-----------------GCTATTGTGTGAGT-ATatttaatatatgat         (-17)

INSERTIONS
409B2      aaatagtgatagatCCATTCCTATGACTGTAGATtttat-cag---actgaagAGCTATTGTGTGAGTATatttaatatatgat      REF
409-LR5a   aaatagtgatagatCCATTCCTATGACTGTAGATtttattcag---actgaagAGCTATTGTGTGAGTATatttaatatatgat      (+1)
```

Supplementary Figure 2. Spectrum of NC-TALEN-induced mutations in human female iPSC clones.

Sequence of *HPRT1* alleles from 409B2 (female) iPSC clones transfected with *HPRT1*_B NC-TALENs and enriched by 6-TG selection on SNL feeders. PCR amplicons of the target site were TA-cloned and at least 8 bacterial colonies from each transformation were PCR-amplified to determine individual alleles by Sanger sequencing. Clones are labeled numerically and alleles alphabetically. iPSC clones with more than two alleles likely represent mosaic populations. Upper case letters represent TALEN binding sites (Fig. 1a). Inserted bases are in italics. Deletion or insertion sizes are indicated on the right. REF, parental 409B2 iPSC reference genomic sequence; NORM, non-mutant allele for the region examined by sequencing.

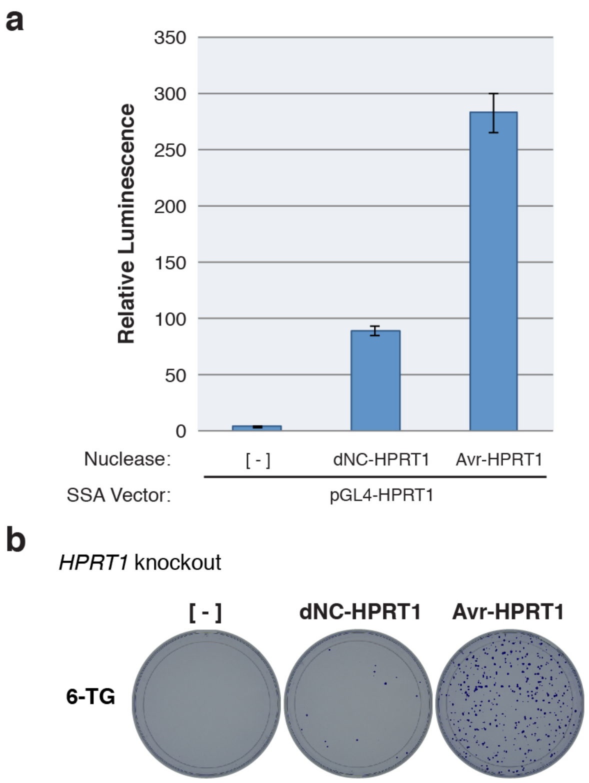

Supplementary Figure 3. Updated TALEN architecture improves *HPRT1*_B cleavage activity.

a. SSA assay comparing the relative activities of *HPRT1*_B TALENs assembled using a PthXo1-based TALE scaffold (NC-TALEN) to an AvrBs3-based +136/+63 scaffold (Avr-TALEN). Error bars show s.e.m. (n = 3).
b. TALEN activity in 1383D6 human male iPSCs as measured by 6-TG^R colony formation, indicating *HPRT1* disruption. Spontaneous colony formation in the absence of nuclease was not noted. For the assay, 3 µg of each nuclease plasmid was transfected into 1 x 10^6 cells by electroporation, followed by plating at a density of 4.5 x 10^5 cells per 60 mm dish. iPSCs were selected and stained as described in the Methods.

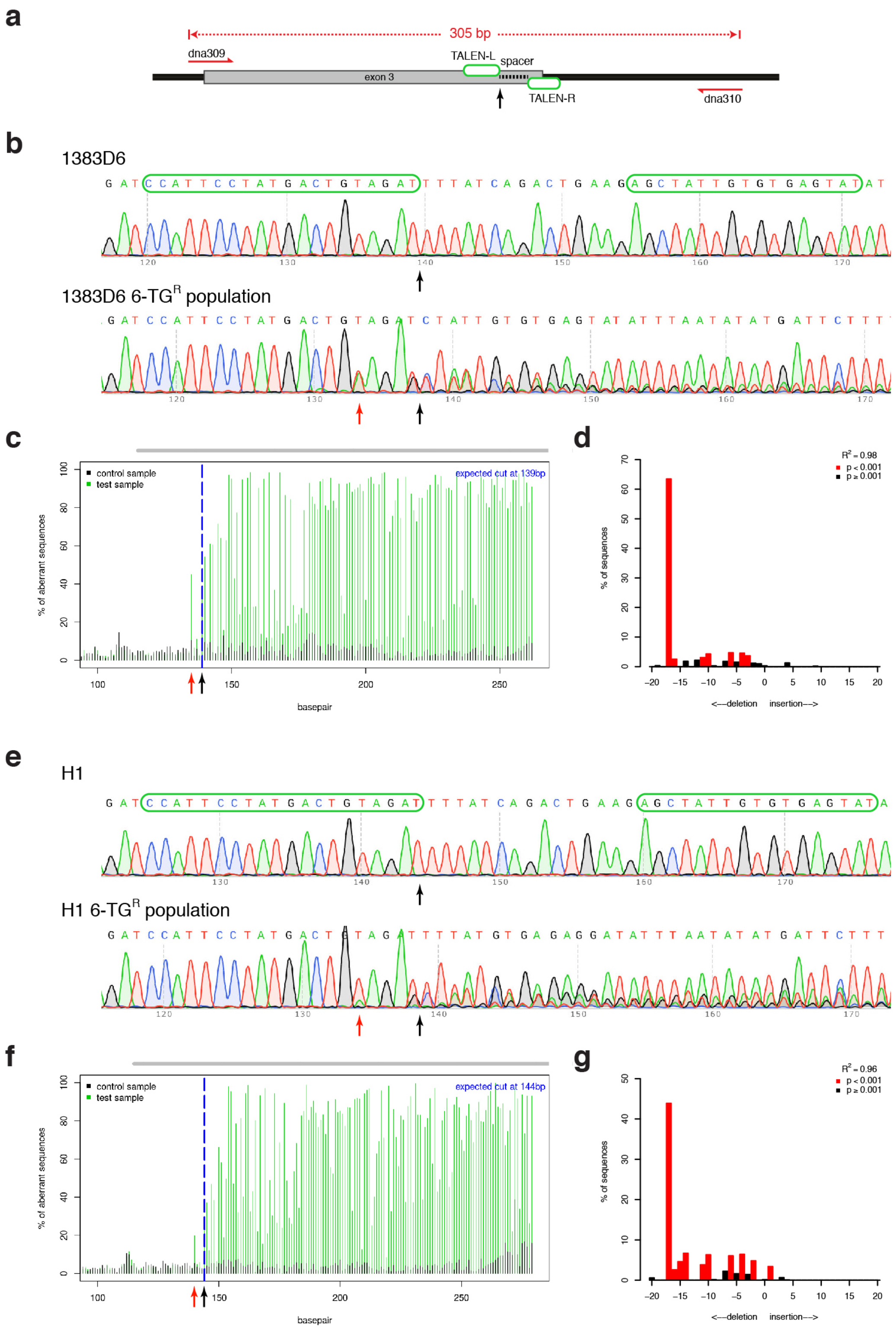

Supplementary Figure 4. TIDE analysis of indel formation at the *HPRT1*_B TALEN target site.

a. Schematic of the genomic PCR assay used to analyze the locus targeted by *HPRT1*_B TALENs. For TIDE analysis, the breakpoint was arbitrarily positioned at the beginning of the spacer as indicated (black arrow).

b. Sequence trace files of the original 1383D6 iPSCs, and 6-TG^R population following transfection with TALENs. The position of the breakpoint used for TIDE analysis is shown (black arrow). An ambiguous A/T base is noted upstream of the predicted breakpoint (red arrow).
c. Aberrant sequence plot determined by the online TIDE software. Arrows are as in Panel b.
d. Spectrum of indels in the 6-TG^R iPSC population as predicted by TIDE. Deletions are more common than insertions, with a clear bias towards 17 bp deletions. The data in Panel c and d was reproduced across independent experiments (n = 3).
e. Sequence trace files of the original H1 ESCs, and 6-TG^R population following transfection with TALENs. The position of the breakpoint used for TIDE analysis is shown (black arrow). An ambiguous A/T base is noted upstream of the predicted breakpoint (red arrow).
f. Aberrant sequence plot determined by the online TIDE software. Arrows are as in Panel e.
g. Spectrum of indels in the 6-TG^R ESC population as predicted by TIDE. As with 1383D6 iPSCs, deletions are more common than insertions, with a clear bias towards 17 bp deletions (n = 1).

DELETIONS

1383D6	aaatagtgatagatCCATTCCTATGACTGTAGATtttatcagactgaagAGCTATTGTGTGAGTATatttaatatatgattctttttag	REF
#2	aaatagtgatagatCCATTCCTATGACTG-----------------aagAGCTATTGTGTGAGTATatttaatatatgattctttttag	(-17,A)
#4	aaatagtgatagatCCATTCCTATGACTG-----------------aagAGCTATTGTGTGAGTATatttaatatatgattctttttag	(-17,A)
#6	aaatagtgatagatCCATTCCTATGACTG-----------------aagAGCTATTGTGTGAGTATatttaatatatgattctttttag	(-17,A)
#7	aaatagtgatagatCCATTCCTATGACTGTAGATt--ctttttag	(-46)
#8	aaatagtgatagatCCATTCCTATGACTGTAGATt--ctttttag	(-46)
#9	aaatagtgatagatCCATTCCTATGACTG-----------------aagAGCTATTGTGTGAGTATatttaatatatgattctttttag	(-17,A)
#11	aaatagtgatagatCCATTCCTATGACTG-----------------aagAGCTATTGTGTGAGTATatttaatatatgattctttttag	(-17,A)
#12	aaatagtgatagatCCATTCCTATGACTGTAGA-----------------GCTATTGTGTGAGTATatttaatatatgattctttttag	(-17,T)
#13	aaatagtgatagatCCATTCCTATGACTGTAGA-----------------GCTATTGTGTGAGTATatttaatatatgattctttttag	(-17,T)
#14	aaatagtgatagatCCATTCCTATGACTGTAGATttt---tag	(-49)
#15	aaatagtgatagatCCATTCCTATGACTGTAGATttta---gactgaagAGCTATTGTGTGAGTATatttaatatatgattctttttag	(-3)
#16	aaatagtgatagatCCATTCCTATGACTGTAGA--------------agAGCTATTGTGTGAGTATatttaatatatgattctttttag	(-14)
#17	aaatagtgatagatCCATTCCTATGACTG-----------------aagAGCTATTGTGTGAGTATatttaatatatgattctttttag	(-17,A)
#21	aaatagtgatagatCCATTCCTATGACTGTAGA-----------------GCTATTGTGTGAGTATatttaatatatgattctttttag	(-17,T)
#24	...--agAGCTATTGTGTGAGTATatttaatatatgattctttttag	(-77)
#25	aaatagtgatagatCCATTCCTATGACTG-----------------aagAGCTATTGTGTGAGTATatttaatatatgattctttttag	(-17,A)
#26	aaatagtgatagatCCATTCCTATGACTG-----------------aagAGCTATTGTGTGAGTATatttaatatatgattctttttag	(-17,A)
#29	aaatagtgatagatCCATTCCTATGACTG-----------------aagAGCTATTGTGTGAGTATatttaatatatgattctttttag	(-17,A)
#30	aaatagtgatagatCCATTCCTATGACTG-----------------aagAGCTATTGTGTGAGTATatttaatatatgattctttttag	(-17,A)
#33	aaatagtgatagatCCATTCCTATGACTG-----------------aagAGCTATTGTGTGAGTATatttaatatatgattctttttag	(-17,A)
#34	aaatagtgatagatCCATTCCTATGACTGTAGA-----------------GCTATTGTGTGAGTATatttaatatatgattctttttag	(-17,T)
#35	aaatagtgatagatCCATTCCTATGACTG-----------------aagAGCTATTGTGTGAGTATatttaatatatgattctttttag	(-17,A)
#36	aaatagtgatagatCCATTCCTATGACTG-----------------aagAGCTATTGTGTGAGTATatttaatatatgattctttttag	(-17,A)
#40	aaatagtgatagatCCATTCCTATGACTGTAGATttt-----------------TTGTGTGAGTATatttaatatatgattctttttag	(-17)
#45	aaatagtgatagatCCATTCCTATGACTG-----------------aagAGCTATTGTGTGAGTATatttaatatatgattctttttag	(-17,A)
#47	aaatagtgatagatCCATTCCTATGACTGTAGATtttatcag--...	(-184)
#48	aaatagtgatagatCCATTCCTATGACTGTAGA-----------------GCTATTGTGTGAGTATatttaatatatgattctttttag	(-17,T)
#49	aaatagtgatagatCCATTCCTATGACTGTAGATt--ctttttag	(-46)
#54	aaatagtgatagatCCATTCCTATGACTG-----------------aagAGCTATTGTGTGAGTATatttaatatatgattctttttag	(-17,A)
#55	aaatagtgatagatCCATTCCTATGACTGTAGATtttat----------------TGTGTGAGTATatttaatatatgattctttttag	(-16)
#56	aaatagtgatagatCCATTCCTATGACTGTAGATtttat----------------TGTGTGAGTATatttaatatatgattctttttag	(-16)

INSERTIONS

1383D6	aaatagtgatagatCCATTCCTATGACTGTAGATtttatcag-----ac----tgaagAGCTATTGTGTGAGTATatttaatatatgattctttttag	REF
#3	aaatagtgatagatCCATTCCTATGACTGTAGATtttatcag*cacag*ac----tgaagAGCTATTGTGTGAGTATatttaatatatgattctttttag	(+5)
#18	aaatagtgatagatCCATTCCTATGACTGTAGATtttatcag-----ac*agac*tgaagAGCTATTGTGTGAGTATatttaatatatgattctttttag	(+4)
#50	aaatagtgatagatCCATTCCTATGACTGTAGATtttat*ttctgtaggactgaacgtcttgctcgagatgtgatgaaggagatgggaggccatca*...	(+156)

COMPLEX

1383D6	cactgaatagaaatagtgatagatCCATTCCTATGACTGTAGATtttatcagactgaagAGCTATTGTGTGAGTATatt	REF
#37	c*tgaagtttaaaaagctaagaggtgtttgttataaagtttaatg*--tatgaaactgaagAGCTATTGTGTGAGTATatt	(-45,+43,C>G, G>A)

Supplementary Figure 5. Spectrum of Avr-TALEN-induced mutations in human male iPSCs clones.

Sequence of *HPRT1* alleles types detected in a series of individual clones derived from 1383D6 iPSC clones transfected with *HPRT1_B* Avr-TALENs and enriched by 6-TG selection under feeder-free conditions. PCR amplicons of the target site were directly Sanger sequenced. Mixed sequences were not included in the analysis. Clones are labeled numerically. Upper case letters represent *HPRT1_B* Avr-TALEN binding sites. Inserted bases are in italics. Modified bases are underlined. Deletion or insertion sizes are indicated on the right. Apart from Δ17, the most common deletion was Δ46 (3/31 deletions), where the deletion boundaries were positioned within T-rich sequences following a predicted 'GATT' microhomology. The Δ77 mutation occurred at another short tandem repeat 'CTGA', again indicative of MMEJ. REF, parental 1383D6 iPSC reference genomic sequence.

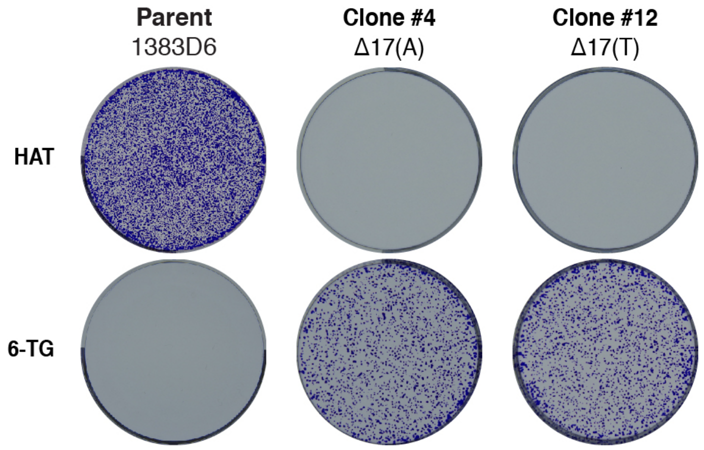

Supplementary Figure 6. Drug sensitivities of 1383D6 parental and *HPRT1* knockout iPSC clones.

Crystal violet staining of representative *HPRT1* knockout clonal iPSC lines following treatment with 6-TG or HAT media for 3 days. Resistance and sensitivity correlates with the status of the *HPRT1* locus, as determined by PCR genotyping and sequencing (Supplementary Fig. 5). Parental 1383D6 iPSCs are included as a control.

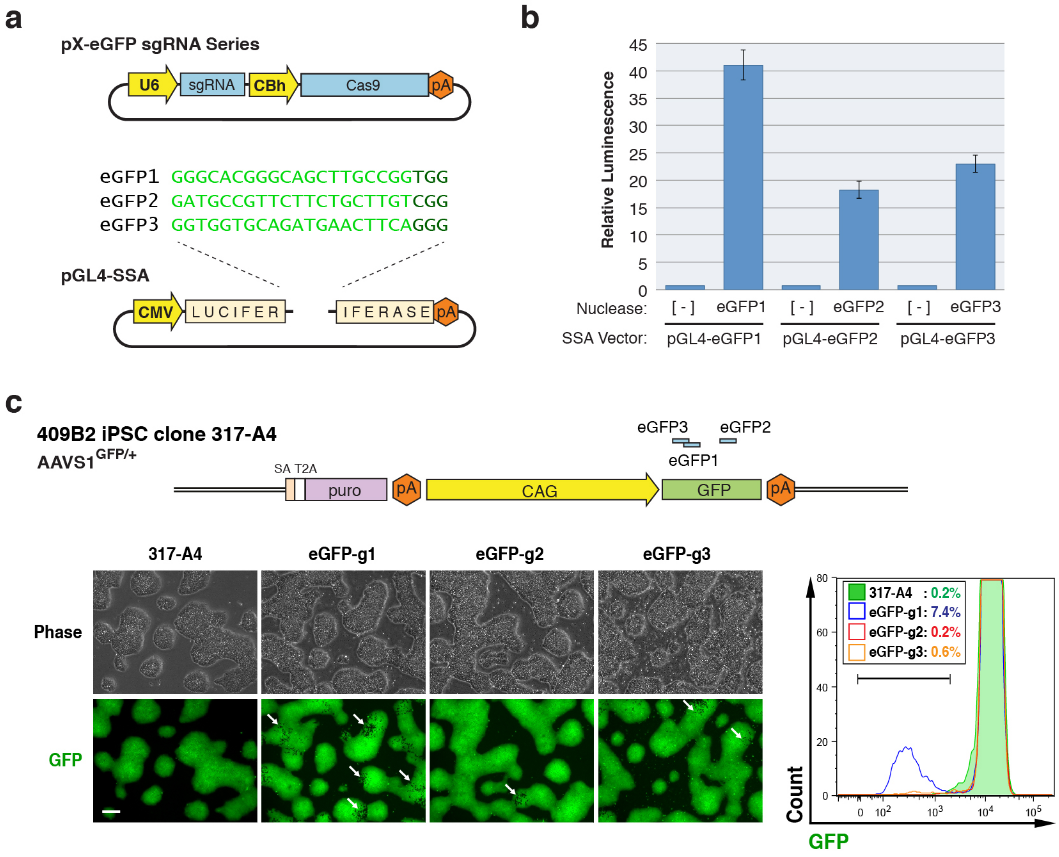

Supplementary Figure 7. Screening eGFP sgRNAs for cleavage activity.

a. Diagram of the sgRNA and Cas9 expression vector pX330 [1], and the associated pGL4-SSA target plasmids used for the plasmid cleavage assay. The three eGFP protospacer sequences [2] are shown.

b. Relative nuclease activities as determined by luciferase expression. pGL4-SSA plasmids were transfected individually with or without the concordant pX-eGFP nuclease plasmid. Error bars show s.e.m. (n = 3).

c. A transgene disruption assay was designed to assess genomic cleavage activity in iPSCs. 317-A4 iPSCs are heterozygous for a constitutively expressed CAG::eGFP reporter transgene targeted to the AAVS1 locus [3]. Relative positions of the three sgRNAs are shown. Microscopy and FACS analysis for GFP expression 5 days after nuclease transfection was used to compare the activities of the three sgRNAs. The most potent sgRNA, eGFP1, is referred to as ‘ps1’ in the Results. White arrows indicate GFP negative regions. Scale bar, 200 µm.

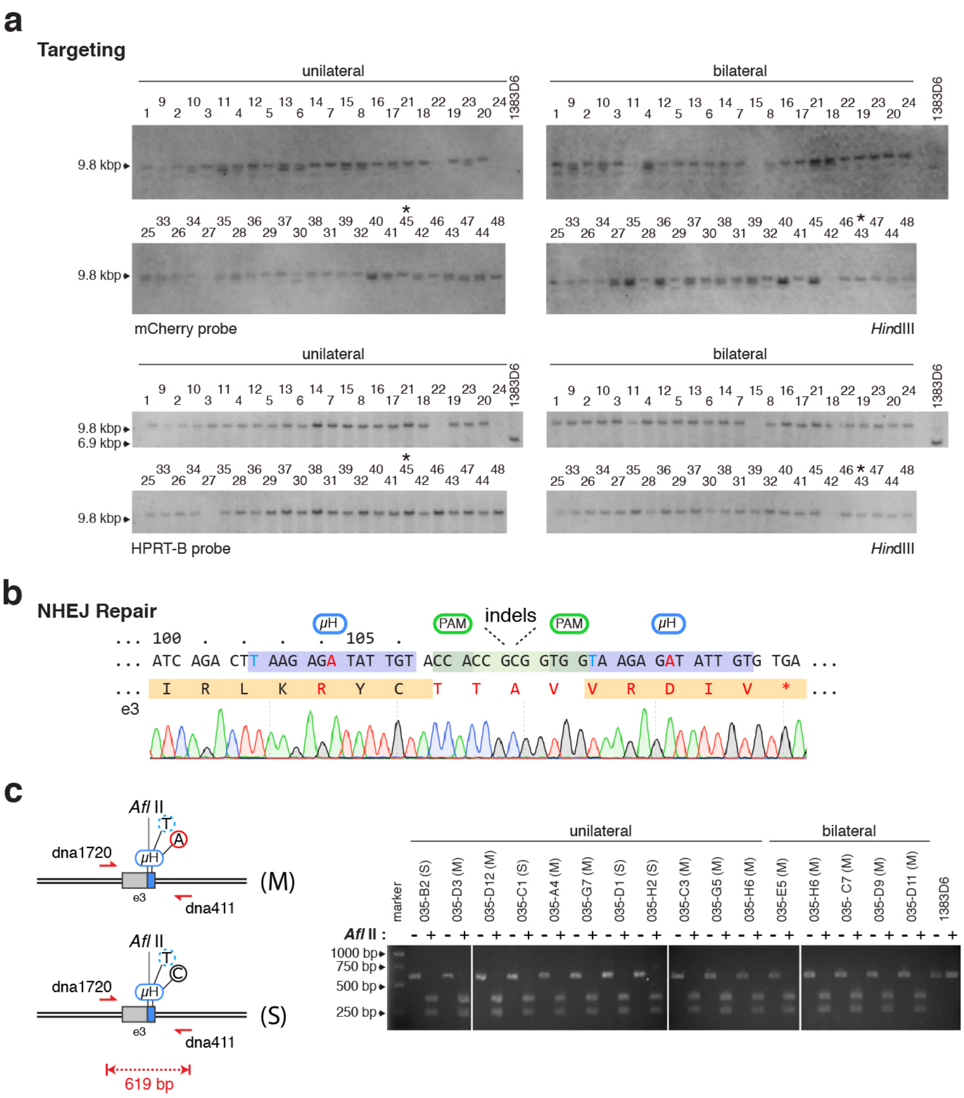

Supplementary Figure 8. Targeting the *HPRT1* locus with cassettes flanked by imperfect microhomology.

a. Southern blotting results for 96 iPSC clones targeted with either unilaterally or bilaterally mutant μH, and probed with either transgene (mCherry, top) or genomic (HPRT-B, bottom) probes. The predicted 6.9 kbp (normal) and 9.8 kbp (targeted) band sizes shown in Fig. 2b are indicated. Selected clones (033-U-45 and 033-B-43) are indicated with an asterisk. 1383D6 iPSCs are included as a control.
b. Sequence trace file of a majority iPSC clone where DSBR following cassette excision is a result of error-free NHEJ. Note direct fusion of the ends predicted to be formed by CRISPR-Cas9-induced DSBs. A minority of these clones included random indels from error-prone NHEJ. Clone proportions are indicated in Table 1.
c. RFLP assay by *Afl*II digestion of PCR amplicons from MhAX iPSC clones engineered with unilateral or bilateral homology, indicating the presence of the engineered Silent (S) mutation in all clones tested. Clones labelled with ‘M’ were found to also contain the Munich mutation by sequencing. 1383D6 iPSCs are included as a negative control for cleavage.

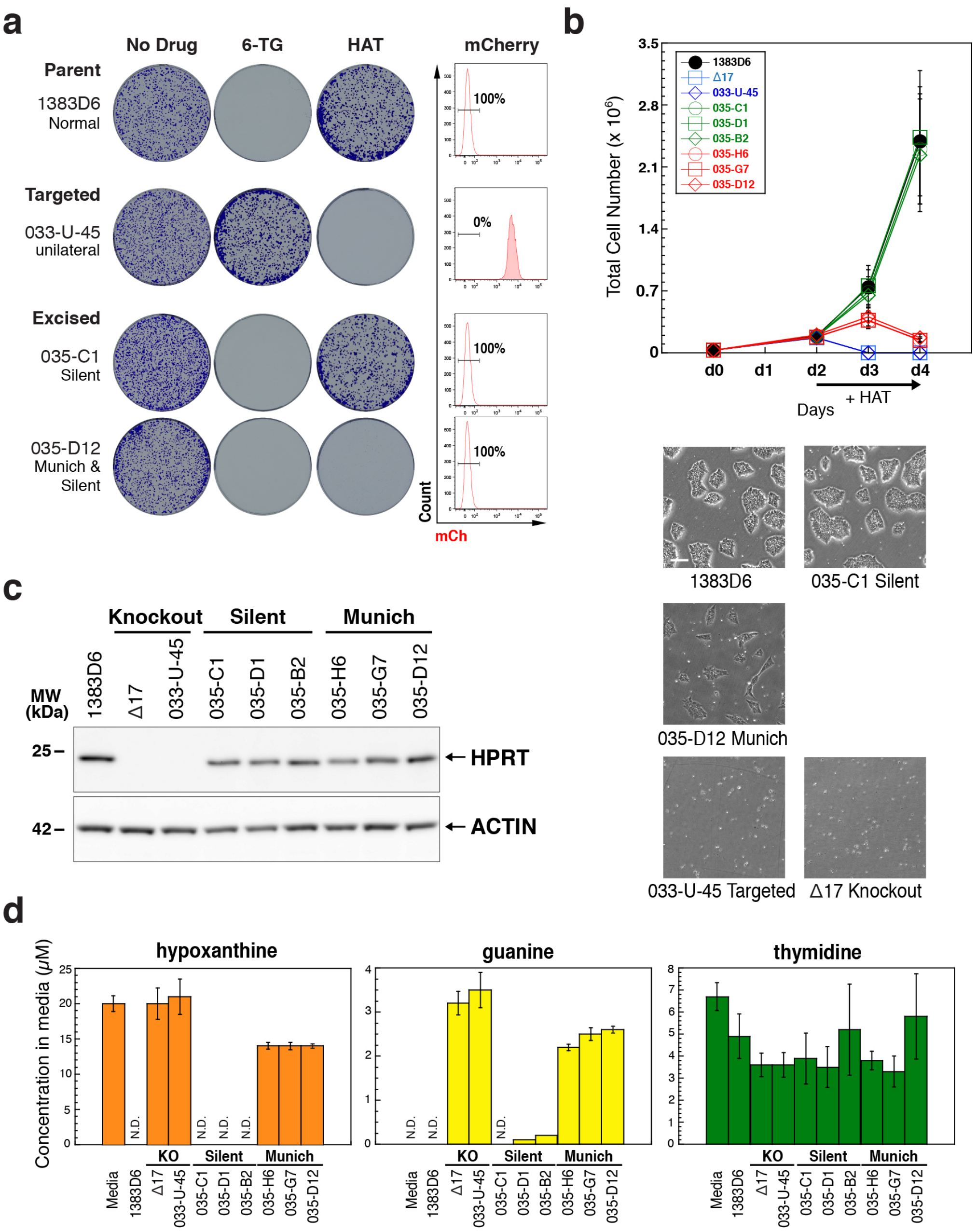

Supplementary Figure 9. Metabolic phenotyping confirms purine salvage defects in $HPRT_{Munich}$ iPSCs.

a. Reversal of 6-TG and HAT drug sensitivities during engineering of the *HPRT1* locus as shown by crystal violet staining of iPSC colonies only occurs for clones with a Silent mutation (035-C1), while clone 035-D12 remains sensitive to both drugs. Original 1383D6 and unilateral parent clone 033-U-45 are included as controls. FACS analysis for mCherry is shown on the right.

b. Growth curve analysis of parental and engineered iPSCs in the presence of HAT selective pressure. $HPRT_{Munich}$ iPSCs show a reduced sensitivity to HAT compared to knockouts (Δ17) or targeted parental clone 033-U-45. The growth of iPSCs with Silent mutations are indistinguishable from 1383D6. Note that the behavior of individual clones with similarly engineered

genotypes were highly comparable. Morphology of iPSCs colonies after 24 hrs of HAT selection is shown below. Image data is representative of two independent experiments. Error bars show s.e.m. (n = 3). Scale bar, 200 µm.

c. Western blot analysis of HPRT protein levels in parental and engineered iPSC clones. Knockout lines Δ17 and 033-U-45 produce no HPRT protein. Expression levels in $HPRT_{Munich}$ and $HPRT_{Silent}$ control clones are comparable to normal 1383D6 iPSCs. ACTIN is used as a loading control.

d. CE-MS metabolite assay of spent media from parental and engineered iPSCs. Hypoxanthine and guanine accumulate as a result of HPRT deficiency, while a partial metabolic defect is observed for $HPRT_{Munich}$ cells. $HPRT_{Silent}$ control iPSCs behave similarly to 1383D6. As expected, thymidine levels are not correlated with *HPRT1* genotype (control). For clones 035-D1 and 035-B2, guanine was detected in only 1 of 3 samples. N.D., not detected. Error bars show s.e.m. (n = 3).

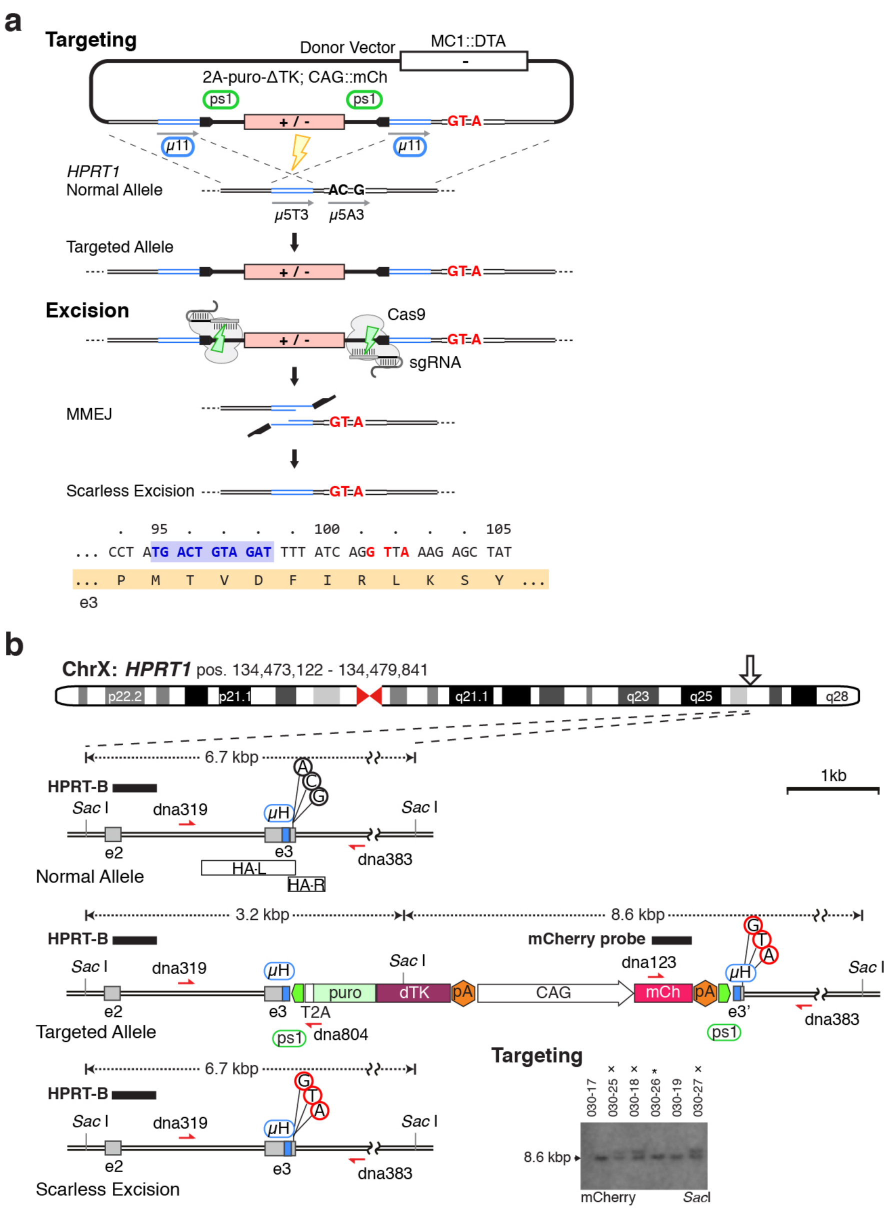

Supplementary Figure 10. Targeting *HPRT1* with a µ11 MhAX cassette.

a. Schematic overview of gene targeting to generate clones for the *HPRT1* chromosomal excision assay. Left and right donor vector homology arms overlap, generating an 11 bp tandem µH (blue) flanking the positive/negative selection marker (red). Synonymous mutations disrupting the endogenous µ5A3 sequence are shown in red. A diphtheria toxin (DTA) negative selection marker driven by the MC1 promoter was included in the donor backbone, but was found to be ineffective (see Panel b, bottom right). Gene targeting was stimulated with Avr*HPRT1_B* TALENs (yellow bolt). The remaining elements are as described in Fig. 2a.
b. Detailed schematic of *HPRT1* gene targeting and MMEJ resolution. Labelling is consistent with Fig. 2b. Southern blot verification of targeted clones using the mCherry probe (bottom right), where an asterisk (*) denotes clones used for subsequent assays (Fig. 3 and Supplementary Fig. 12) while “x” indicates clones with random integration.

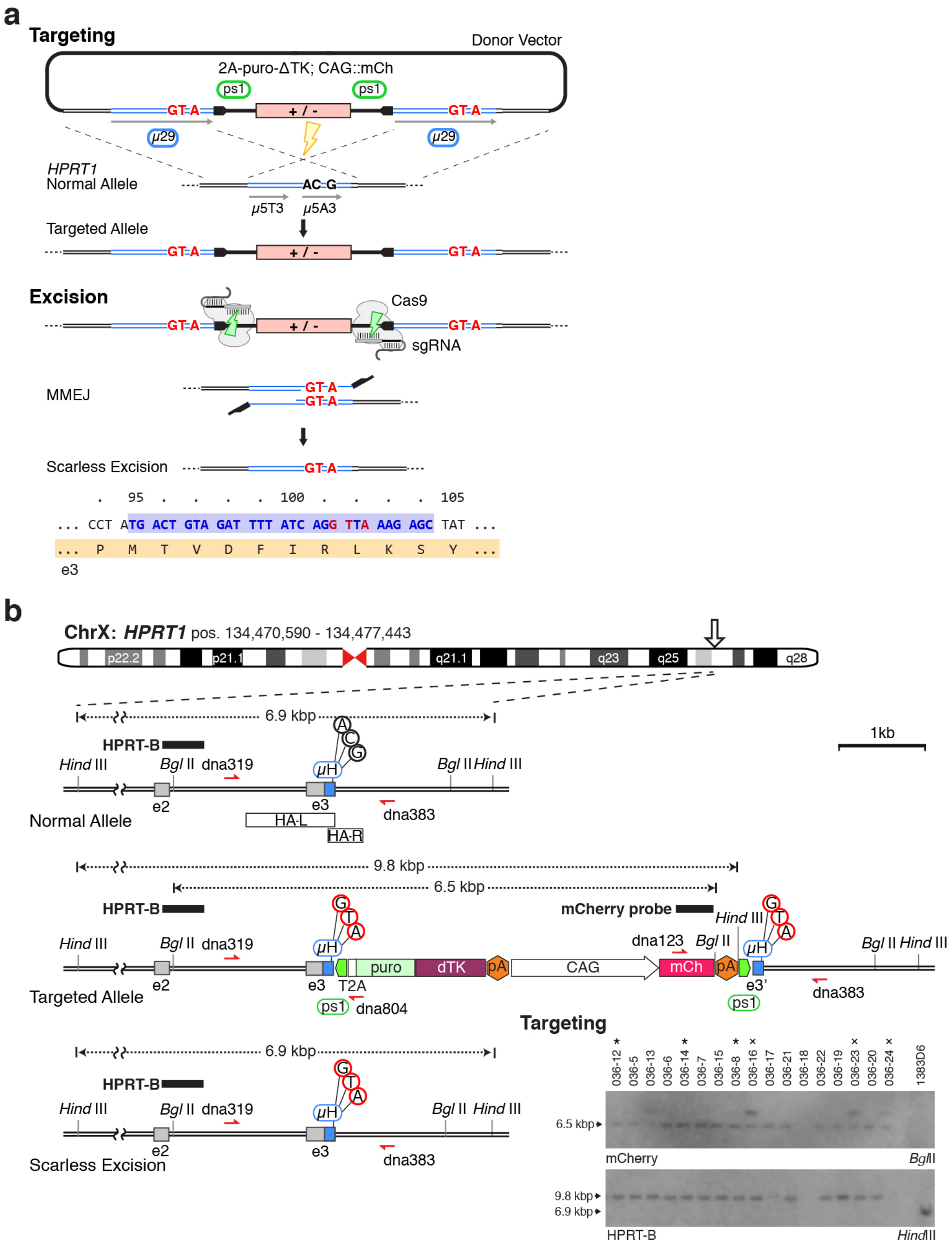

Supplementary Figure 11. Targeting *HPRT1* with a μ29 MhAX cassette.

a. Schematic overview of gene targeting to generate clones for the *HPRT1* chromosomal excision assay. Left and right donor vector homology arms overlap, generating a 29 bp tandem μH (blue) flanking the positive/negative selection marker (red). Synonymous mutations disrupting the endogenous μ5A3 sequence are shown in red. Gene targeting was stimulated with Avr*HPRT1_B* TALENs (yellow bolt). The remaining elements are as described in Fig. 2a.

b. Detailed schematic of *HPRT1* gene targeting and MMEJ resolution. Labelling is consistent with Fig. 2b. Southern blot verification of targeted clones using the mCherry and HPRT-B probes (bottom right), where an asterisk (*) denotes clones used for subsequent assays (Fig. 3, Table 2 and Supplementary Fig. 12) while "x" indicates clones with random integration.

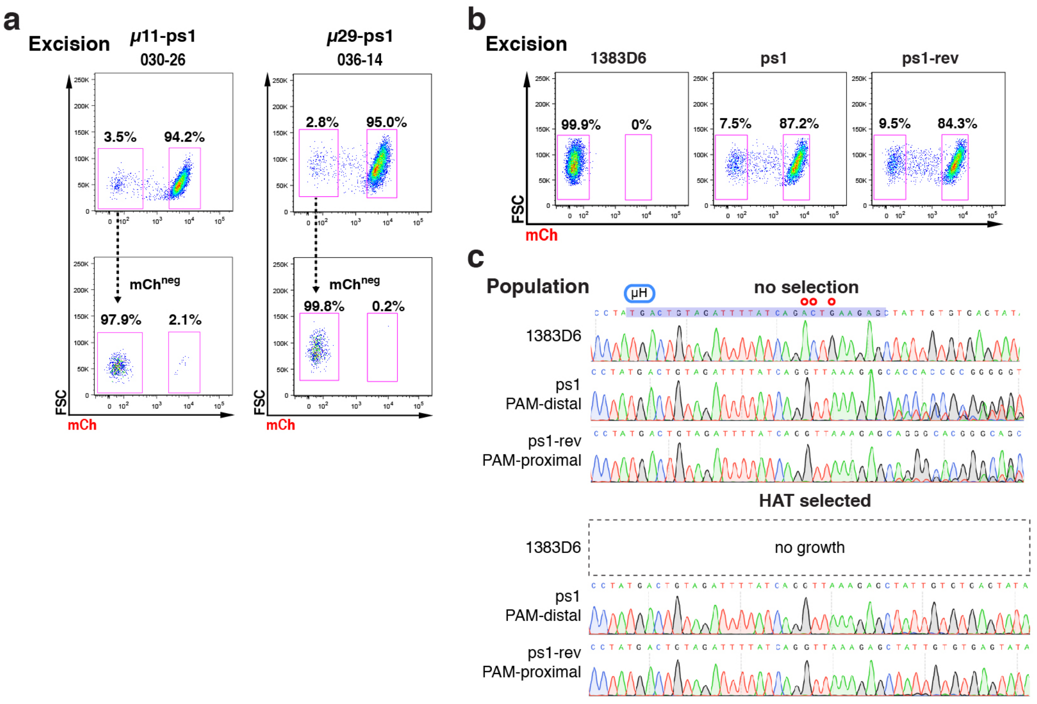

Supplementary Figure 12. Effect of protospacer inversion on MMEJ repair.

a. FACS for mCh^{neg} cells following transfection of targeted iPSC clones (differing in μH length) with pX-ps1 to stimulate cassette excision. μ29 excision data is representative of three independent clones.

b. FACS analysis for mCh^{neg} cells following transfection of targeted iPSC clones (inverted protospacers) with pX-ps1. Parental 1383D6 iPSCs are included as a control. Clones for this assay were generated using gene targeting as outlined in Supplementary Fig. 11, except with inverted ps1 protospacers in the case of ps1-rev.

c. Sanger sequencing of excised populations shown in Panel b with and without HAT selection. With HAT selection, the predominance of indel-free sequences bearing engineered synonymous mutations indicates that the population is biased towards MMEJ repair, irrespective of the ps1 protospacer orientation. μH regions (blue) and synonymous mutations (red) are indicated.

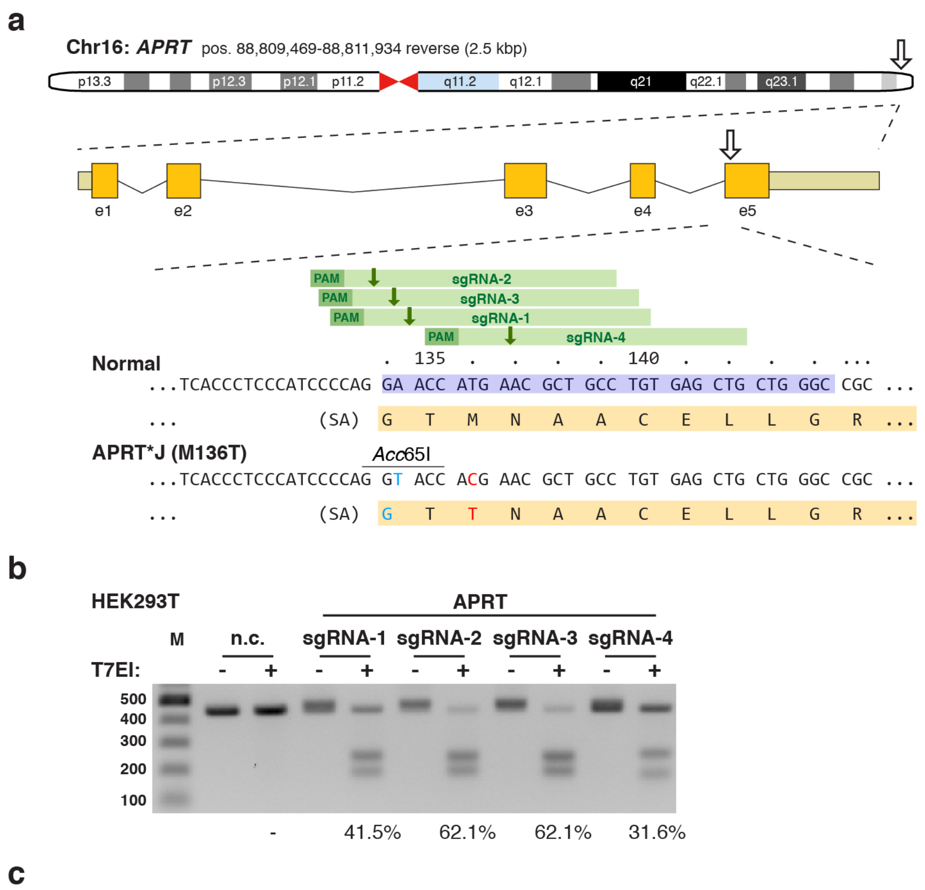

iPSC (1383D6)	n.c.	sgRNA-1	sgRNA-2	sgRNA-3	sgRNA-4
puro^R colonies:	0	71	626	127	21

Supplementary Figure 13. Validation of APRT sgRNAs.

a. Schematic of the human *APRT* locus and strategy for engineering the APRT*J mutation. Detail is shown for exon 5 (orange) including the splice junction, CRISPR-Cas9 target sites 1 through 4 (green), and selected µ32 microhomology (blue). *APRT* codons are numbered above. Chromosome positions refer to *H. sapiens* GRCh38. Bases targeted for MhAX editing are shown in blue (silent) or red (APRT*J). SA, splice acceptor.
b. T7EI assay results revealing the activity of sgRNAs 1 through 4 in HEK293T cells. n.c., negative control without nuclease transfection.
c. Puro^R iPSC colony numbers resulting from *APRT* gene targeting stimulated with sgRNAs 1 through 4. One million 1383D6 iPSCs were electroporated with 3 µg of APRT-2A-puroΔTK donor vector only (n.c.), or the donor plus 1 µg of the appropriate sgRNA expression vector and plated on two 60 mm dishes (5 x 10^5 cells each). Colony numbers are the total from two dishes.

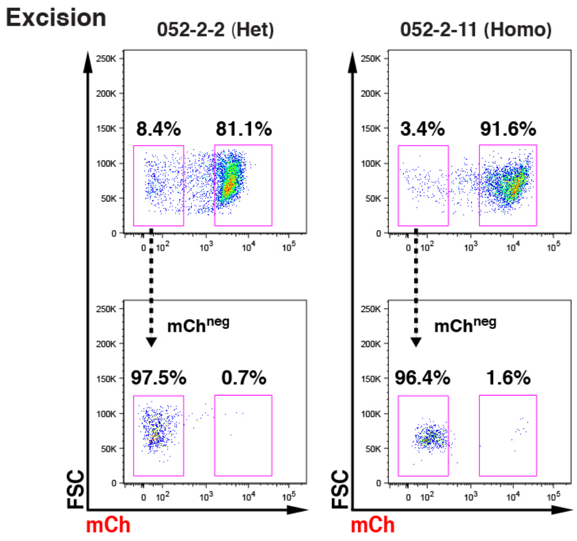

Supplementary Figure 14. Flow cytometry analysis of *APRT* gene targeting and excision.

FACS for mCh^{neg} cells following transfection with pX-ps1 to stimulate cassette excision. As expected, excision rates are lower for homozygously targeted clones.

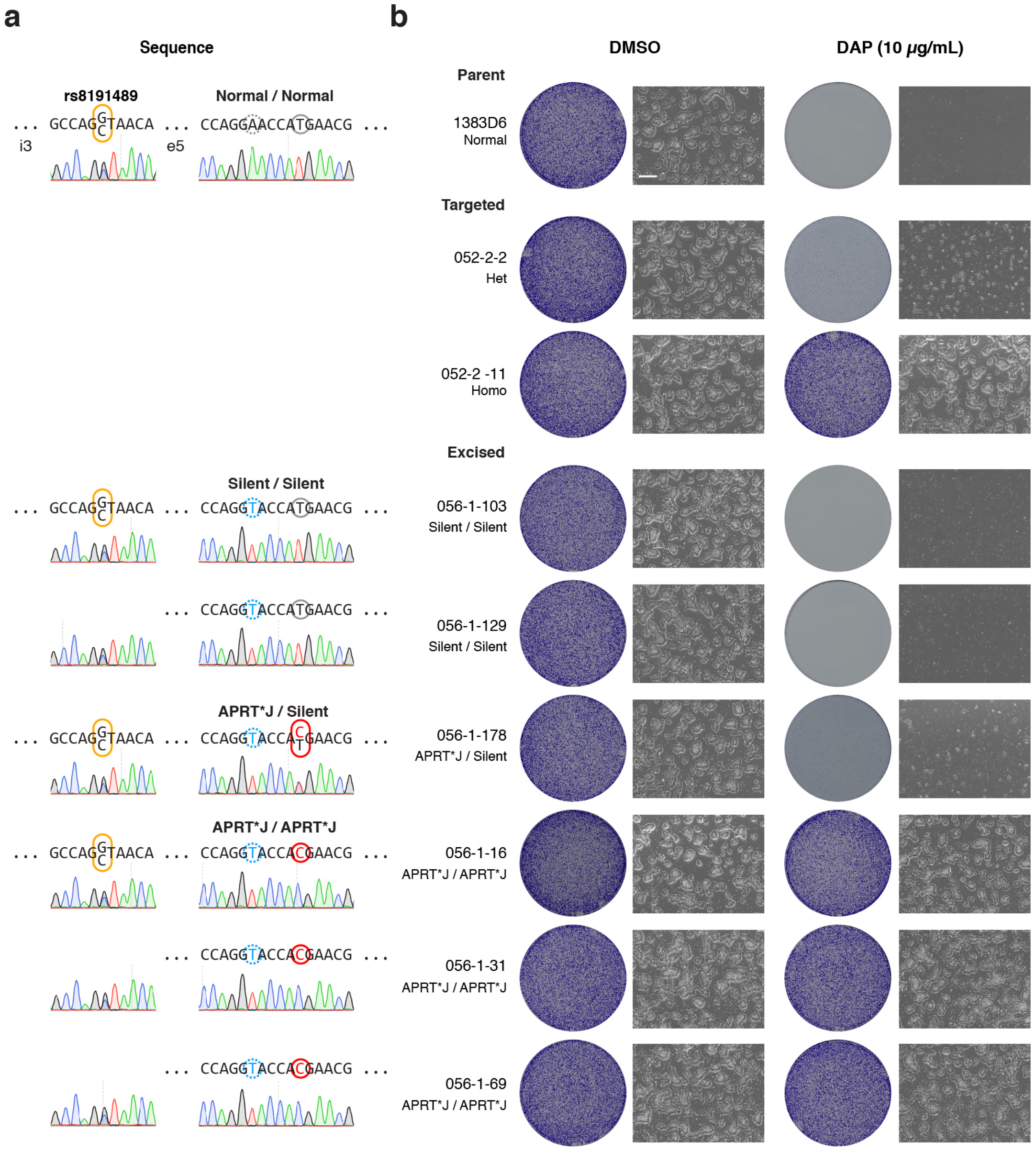

Supplementary Figure 15. Metabolic phenotyping confirms altered enzyme function in mono- and biallelically modified APRT*J iPSCs.

a. Sequence trace files of iPSC clones biallelically engineered with APRT*J and/or Silent mutations following scarless MMEJ cassette excision. Both types of clones were isolated from the same targeted iPSC (052-2-11). Inclusion of the neighboring heterozygous SNP (rs8191489) in the PCR amplicon ensures analysis of both alleles.
b. Crystal violet staining of iPSC culture dishes following treatment with DMSO (left), or DAP (right) for a period of 2 d. Scale bar, 500 µm.

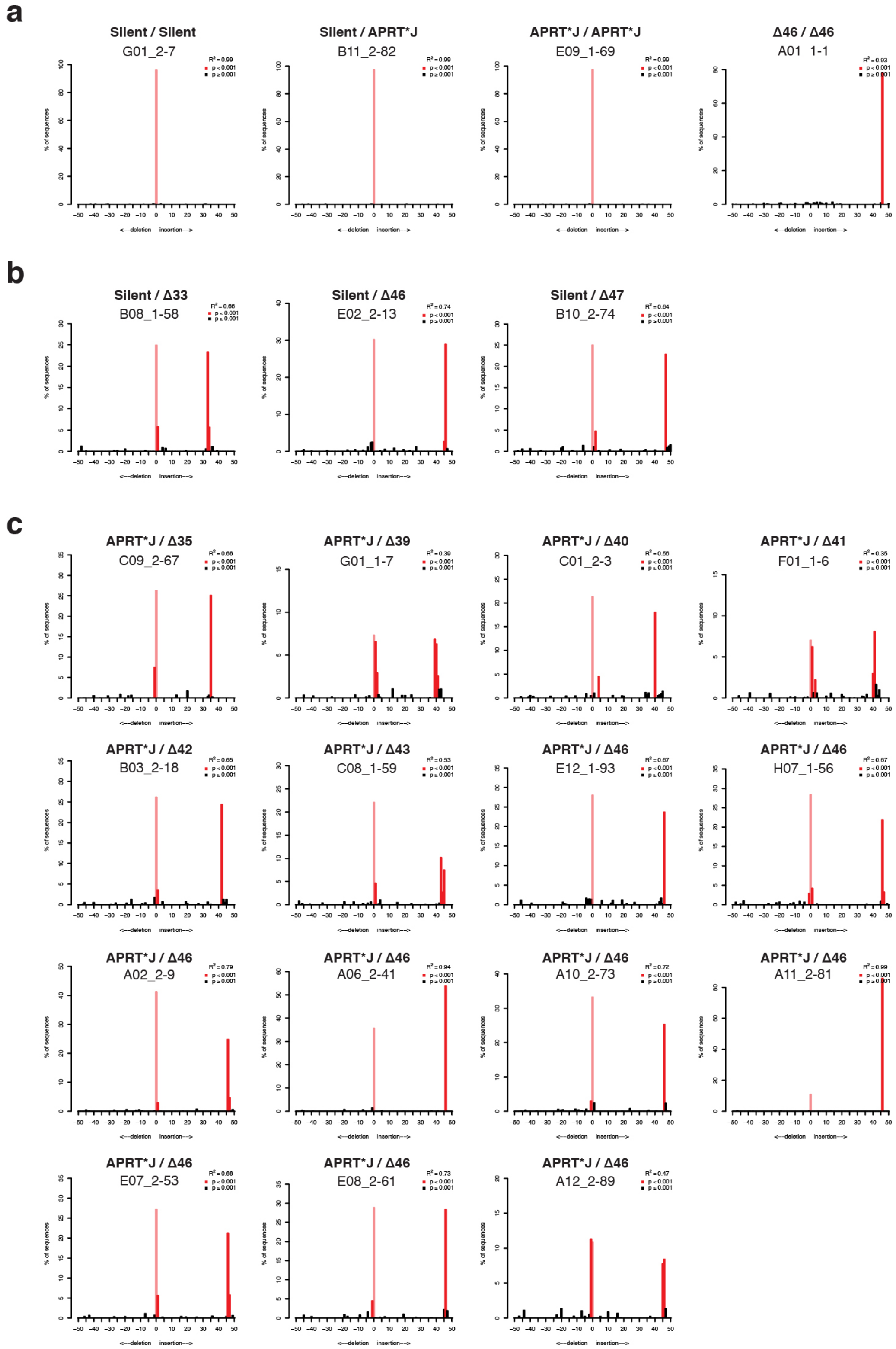

Supplementary Figure 16. TIDE analysis of biallelically repaired iPSC clones.

a. Representative TIDE analysis for biallelic repair of the *APRT* locus by MMEJ (Silent/Silent, Silent/APRT*J, APRT*J/APRT*J) or perfect NHEJ (Δ46/Δ46).

b. Representative TIDE analysis for biallelic repair of the *APRT* locus by two different DSBR mechanisms; MMEJ resulting in deposition of a Silent point mutation on one allele, and NHEJ resulting in a random indel on the other.
c. Representative TIDE analysis for biallelic repair of the *APRT* locus by two different DSBR mechanisms; MMEJ resulting in deposition of APRT*J & Silent point mutations on one allele (APRT*J), and NHEJ resulting in a random indel on the other. Genotypes listed in Panels a-c were verified by sequence alignment to the reference human genome.

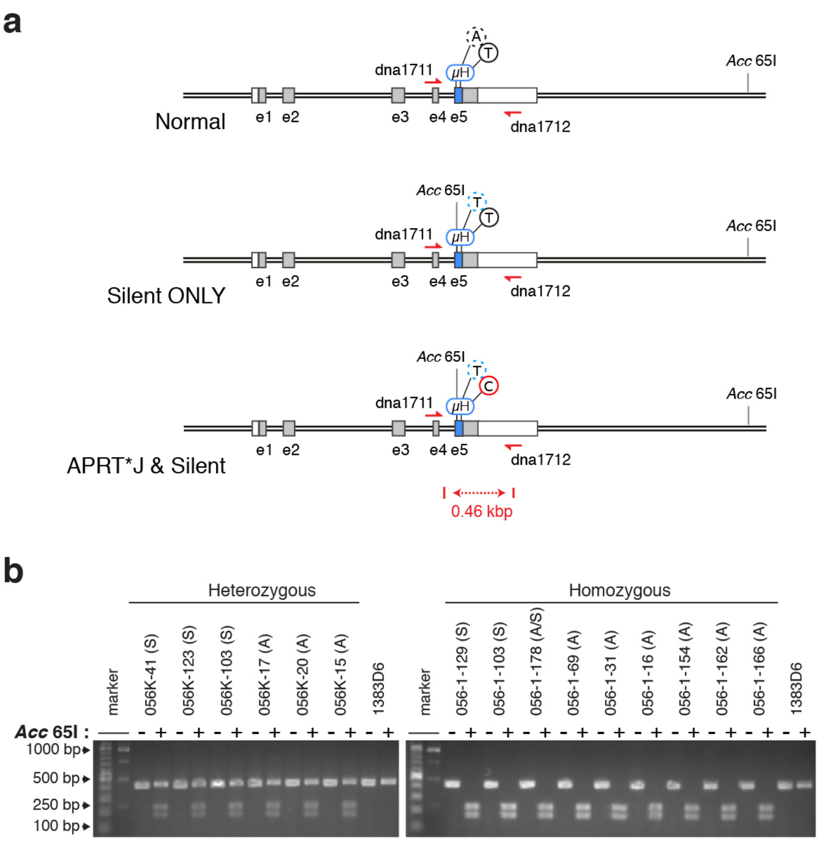

Supplementary Figure 17. RFLP assay for the *APRT* Silent mutation.

a. Schematic of the parental and edited *APRT* alleles, and the resulting RFLP generated by the Silent mutation.
b. Gel electrophoresis following *Acc*65I digestion of PCR amplicons from excised hetero- or homozygously targeted iPSC clones, indicating the presence of the engineered Silent mutation. 1383D6 iPSCs are included as a negative control for cleavage.

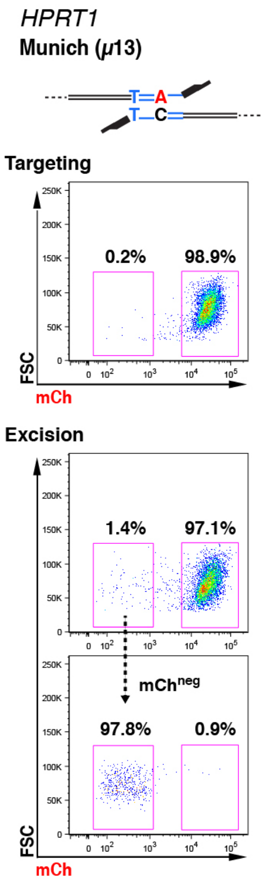

Supplementary Figure 18. FACS-based isolation of edited HPRT$_{\text{Munich}}$ iPSCs. Representative FACS plots for the isolation of iPSCs edited at the *HPRT1* locus. The donor vector, allele, and additional features are as described in Fig. 2a and b.

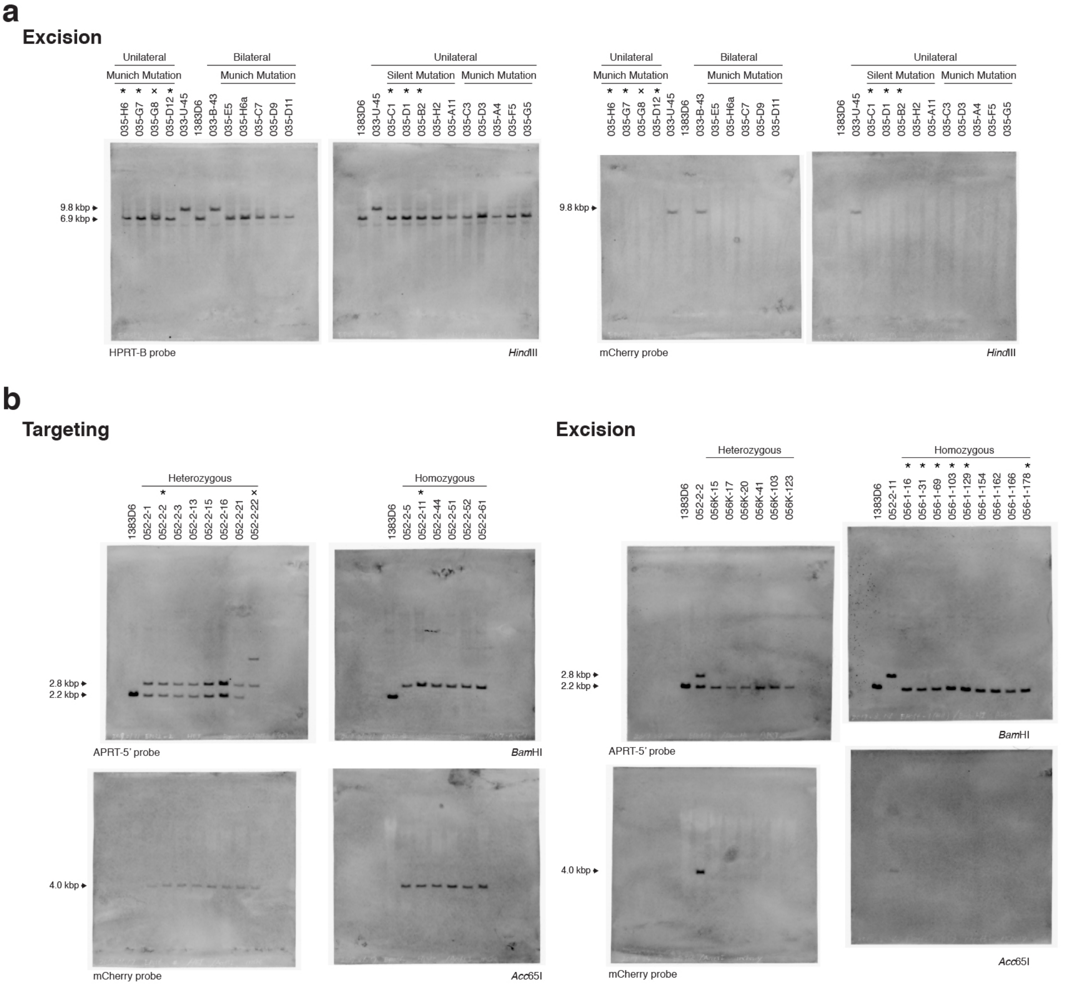

Supplementary Figure 19. Uncropped Southern blot images.

a. Complete images for Southern blot genotyping data shown in Fig. 2d.
b. Complete images for Southern blot genotyping data shown in Fig. 4c and f.

Supplementary Tables

Supplementary Table 1. Characteristics of engineered microhomologies used in this study

Purpose	Name	Mutation	Pos.	Laterality	µH Sequence *	Len.	GC (%)	PAM +1	Het.
HPRT-Native	µ5W3	T	5'	uni	GACTGtAGA	9	44	n/a	≦ 8
		A	3'		GACTGaAGA	9	44	n/a	≦ 8
HPRT Munich	µ13	Munich, Silent	5', 3'	bi	tAAGAGaTATTGT	13	23	T	7, 6
	µ13 (µ6M6)	Munich, Silent	5'	uni	tAAGAGaTATTGT	13	23	T	7
		Silent	3'		tAAGAGCTATTGT	13	31	T	6
MMEJ Assay (Plasmid)	µ5	none	5', 3'	bi	CGAGG	5	80	C	7
	µ10	syn	5', 3'	bi	CGAGGCTAAa	10	50	C	7
	µ15	syn	5', 3'	bi	CGAGGCTAAaGTcGT	15	53	C	7
	µ20	syn	5', 3'	bi	CGAGGCTAAaGTcGTtGAtT	20	45	C	7
	µ30	syn	5', 3'	bi	CGAGGCTAAaGTcGTtGAtTTGGACACCGG	30	53	C	7
	µ40	syn	5', 3'	bi	CGAGGCTAAaGTcGTtGAtTTGGACACCGGTAAGACACTg	40	50	C	6, 7
	µ50	syn	5', 3'	bi	CGAGGCTAAaGTcGTtGAtTTGGACACCGGTAAGACACTgGGTGTGAACC	50	52	C	7
MMEJ Assay (HPRT)	µ11	syn (external)	5', 3'	bi	TGACTGTAGAT	11	36	T	7, 6
	µ29	syn	5', 3'	bi	TGACTGTAGATTTTATCAGgtTaAAGAGC	29	34	T	7, 6
		syn	5', 3'	bi	TGACTGTAGATTTTATCAGgtTaAAGAGC	29	34	A	18, 17
APRT*J	µ6Y25	APRT*J, Silent	5'	uni	GtACCAcGAACGCTGCCTGTGAGCTGCTGGGC	32	66	A	7
		Silent	3'		GtACCATGAACGCTGCCTGTGAGCTGCTGGGC	32	63	A	7

* Lower-case characters indicate mutations. Pos., position; Len., length; Het., heterology; Syn, synonymous mutation; uni, unilateral; bi, bilateral.

Supplementary Table 2. HPRT allele spectrum following FACS enrichment

	non-targeted		NHEJ	MMEJ		
**Samples Analyzed**	**Normal Allele**	**indel**	**NHEJ (Perfect)**	**Silent ONLY**	**Munich & Silent**	**Fidelity (%)**
90	0	1	84 (36)	2	3	5.6

Supplementary Table 3. Plasmids used in this study

Purpose	Plasmid ID #	Plasmids
TALENs	KW228	PB-CAG-dNC-HPRT1_L-GFP
	KW229	PB-CAG-dNC-HPRT1_R-mCh
	TY026	CAG-Avr-HPRT-LEFT
	TY027	CAG-Avr-HPRT-RIGHT
CRISPR/Cas9	KW532	pX-EGFP-g1 (alias: pX-ps1)
	KW533	pX-EGFP-g2
	KW534	pX-EGFP-g3
	KW817	pX-APRT-sg1
	KW818	pX-APRT-sg2
	KW819	pX-APRT-sg3
	KW820	pX-APRT-sg4
HPRT Donor Vectors	KW293	p3-HPRT1
	KW836	p3-HPRT1-S104R-PdTK-mCh
	KW838	p3-HPRT1-S104Rf-PdTK-mCh
	KW794	pDT-HPftsG1-CAG-mCh
	KW883	pHPftsG1-u29-CAG-mCh
	KW1033	pbG-HPRT-u29-eGFP1-PdTKmCh
	KW1034	pbG-HPRT-u29-eGFP1rev-PdTKmCh
APRT Donor Vectors	KW827	pCR4-hAPRT-G
	KW1005	pbG-APRT-J-u32uni-PdTKmCh
Donor Cassette	KW999	pAAVS1-PdTK-CAG-mCh-[uBglII]
Donor Backbone	KW991	pCAG-eGFP-pA
SSA assay (luciferase)	KW206	pGL4-AAVS1
	KW850	pGL4-SSA-eGFP1
	KW859	pGL4-SSA-eGFP2
	KW862	pGL4-SSA-eGFP3
MMEJ assay (luciferase)	KW855	pGL4K-MMEJ-eGFP1-µ0
	KW868	pGL4K-MMEJ-eGFP1- µ5
	KW856	pGL4K-MMEJ-eGFP1- µ10
	KW869	pGL4K-MMEJ-eGFP1- µ15
	KW857	pGL4K-MMEJ-eGFP1- µ20
	KW858	pGL4K-MMEJ-eGFP1- µ30
	KW875	pGL4K-MMEJ-eGFP1- µ40
	KW876	pGL4K-MMEJ-eGFP1- µ50
Luciferase Assay Controls	KW208	pGL4-CMV-luc2
	Promega E6921	pGL4_74_hRlucTK

Supplementary Table 4. Primers used for donor vector construction in this study

Gene	Purpose	Primer ID#	Primer Name	Sequence	Product Size (bp)
HPRT	Homology Region	dna450	hHPRT-Fo	GTGCAGTGCAGCAGAATGAT	1253
		dna411	hHPRT1Cel-Rev2	ATTTGTCAAACCTAGCTCCAAAGG	
	KW794 InFusion	dna1649	HPRT-Ifs	CTCTATGGGTCGAC*GGGCACGGGCAGCTTGCCGGTGG*TGACTGTAGATTTTATCAGgtTaAAGAGCTATTGTGTGAGTAT	3717
		dna1644	HPRT-Ifas	ACTTCCTCTGCCCTC*GGGCACGGGCAGCTTGCCGGTGG*TATCTACAGTCATAGGAATGGATCTATCACTATTTCT	
	KW836, KW838 InFusion	dna1714	Munich-IF-R (common)	ACTTCCTCTGCCCTC*GGGCACGGGCAGCTTGCCGGTGG*TACAATAtCTCTTaAGTCTGATAAAATCTACA	3713
		dna1713	Munich-IF-F (unilateral)	CTCTATGGGTCGAC*GGGCACGGGCAGCTTGCCGGTGG*tAAGAGCTATTGTGTGAGTATATTTAATATATG	
		dna1715	Munich-flank-IF-F (bilateral)	CTCTATGGGTCGAC*GGGCACGGGCAGCTTGCCGGTGG*tAAGAGaTATTGTGTGAGTATATTTAATATATG	
	KW883 InFusion	dna1649	HPRT-Ifs	CTCTATGGGTCGAC*GGGCACGGGCAGCTTGCCGGTGG*TGACTGTAGATTTTATCAGgtTaAAGAGCTATTGTGTGAGTAT	3737
		dna1864	HPRT-u30-Ifas	ACTTCCTCTGCCCTC*GGGCACGGGCAGCTTGCCGGTGG*TGCTCTTtAacCTGATAAAATCTACAGTCATAGGAATG	
	2A-puro-delTK InFusion	dna1642	T2A-pdtk-Fo	GAGGGCAGAGGAAGTCTTCTAACAT	1930
		dna1643	T2A-pdtk-Rev	GTCGACCCATAGAGCCCACCG	
	KW1033, KW1034 InFusion	dna2167	HPRTCommon-Acc-A	GCGAATTGGGTACCGTGCAGTGCAGCAGAATGATCAC	946
		dna2169	u29-eGFP1-B	TCCGCTGCCAGATCT*GGGCACGGGCAGCTTGCCGGTGG*TGCTCTTtAacCTGATAAAATCTACAGTCATAGGAATGGATC	
		dna2171	u29-eGFP1rev-B	TCCGCTGCCAGATCT*CCACCGGCAAGCTGCCCGTGCCC*TGCTCTTtAacCTGATAAAATCTACAGTCATAGGAATGGATC	
		dna2170	u29-eGFP1-C	TGCAGCCCAAGCTT*GGGCACGGGCAGCTTGCCGGTGG*TGACTGTAGATTTTATCAGgtTaAAGAGCTATTGTGTGAGTAT	442
		dna2172	u29-eGFP1rev-C	TGCAGCCCAAGCTT*CCACCGGCAAGCTGCCCGTGCCC*TGACTGTAGATTTTATCAGgtTaAAGAGCTATTGTGTGAGTAT	
		dna2168	HPRTCommon-Acc-D	CATCATGGCCGGTACCCATTTGTCAAACCTAGCTCCAAAGGACT	
APRT	Homology Region	dna1692	hAPRT-HAF	ACTCCTGTCACTTACCCTGA	1255
		dna1695	hAPRT-HAR	CTGGAGGGTTCTAGCTCCTG	
	KW1005 InFusion	dna2163	APRT-Acc65I-A	GCGAATTGGGTACcACTCCTGTCACTTACCCTGACAGGCCTAG	825
		dna2164	APRT-J-Acc-B	CTCCGCTGCCAGATCT*GGGCACGGGCAGCTTGCCGGTGG*aGCCCAGCAGCTCACAGGCAGCGTTCgTGGTaCCTGGGGATGGGAGGGTGA	
		dna2165	APRT-Acc-C	CCTGCAGCCCAAGCTT*GGGCACGGGCAGCTTGCCGGTGG*aGtACCATGAACGCTGCCTGTGAG	570
		dna2166	APRT-Acc65I-D	TCATGGCCGGTACCCTGGAGGGTTCTAGCTCCTGAGGTG	

Operational sequences in MhAX InFusion primers are annotated as follows: underline, InFusion homology; italics, ps1 (eGFP1) protospacer; bold italics, PAM; double underline, microhomology; lowercase, mutations.

Supplementary Table 5. Primers used for sgRNA construction in this study

Target	sgRNA	Primer ID#	Primer Name	Sequence
eGFP	eGFP-1	dna1045	EGFP-gRNA1-Fo	caccgGGGCACGGGCAGCTTGCCGG
		dna1046	EGFP-gRNA1-Rev	aaacCCGGCAAGCTGCCCGTGCCCc
	eGFP-2	dna1047	EGFP-gRNA2-Fo	caccgGATGCCGTTCTTCTGCTTGT
		dna1048	EGFP-gRNA2-Rev	aaacACAAGCAGAAGAACGGCATCc
	eGFP-3	dna1049	EGFP-gRNA3-Fo	caccgGGTGGTGCAGATGAACTTCA
		dna1050	EGFP-gRNA3-Rev	aaacTGAAGTTCATCTGCACCACCc
APRT	APRT-sg1	dna1678	APRT-Xs1	caccgCAGGCAGCGTTCATGGTTCC
		dna1679	APRT-Xas1	aaacGGAACCATGAACGCTGCCTGc
	APRT-sg2	dna1680	APRT-Xs2	caccgGGCAGCGTTCATGGTTCCTG
		dna1681	APRT-Xas2	aaacCAGGAACCATGAACGCTGCCc
	APRT-sg3	dna1682	APRT-Xs3	caccgAGGCAGCGTTCATGGTTCCT
		dna1683	APRT-Xas3	aaacAGGAACCATGAACGCTGCCTc
	APRT-sg4	dna1684	APRT-Xs4	caccgCAGCTCACAGGCAGCGTTCA
		dna1685	APRT-Xas4	aaacTGAACGCTGCCTGTGAGCTGc
Sequence validation		dna790	U6-fwd	GAGGGCCTATTTCCCATGATTCC

Lower-case characters indicate overhangs for BbsI cloning and 5'-G.

Supplementary Table 6. Primers used for luciferase vector construction in this study

Assay	Purpose	Primer ID#	Primer Name	Sequence
SSA Assay	SSA-AAVS1	dna199	AAVS1-SSAfo	gtcgGATATCTGTCCCCTCCACCCCACAGTGGGGCCACTAGGGACAGGATTGGTGACAGAAAAGCCCCAGGT
		dna200	AAVS1-SSArev	cggtACCTGGGGCTTTTCTGTCACCAATCCTGTCCCTAGTGGCCCCACTGTGGGGTGGAGGGGACAGATATC
	SSA-eGFP-1	dna1804	eGFP1-SSAs	gtcgGGGCACGGGCAGCTTGCCGGTGG
		dna1805	eGFP1-SSAas	cggtCCACCGGCAAGCTGCCCGTGCCC
	SSA-eGFP-2	dna1806	eGFP2-SSAs	gtcgGATGCCGTTCTTCTGCTTGTCGG
		dna1807	eGFP2-SSAas	cggtCCGACAAGCAGAAGAACGGCATC
	SSA-eGFP-3	dna1808	eGFP3-SSAs	gtcgGGTGGTGCAGATGAACTTCAGGG
		dna1809	eGFP3-SSAs	cggtCCCTGAAGTTCATCTGCACCACC
	Sequence validation	dna197	SSAseq-Fo	CTCAGCAAGGAGGTAGGTGAGG
		dna198	SSAseq-Rev	TGATCGGTAGCTTCTTTTGCAC
MMEJ Assay	ccdB Cassette (µH 0-30 bp)	dna1842	CamccdB-F	GGATCCGGTACCGAATTCGCGGCCGCATTAGGCAC
		dna1843	CamccdB-R	GCGGCCGCGAATTCtGTCGACCTGCAGACTGGCTGTG
	Common (µH 0-30 bp)	dna1828	luc2-eGFP1-uH-F	AGAATTCGCGGCCGC*GGGCACGGGCAGCTTGCCGG****TGG***cCGAGGCTAAaGTcGTtGAtTTGGACACCGGTAAGACACTGGGT
	µ0	dna1821	luc2-eGFP1-u0-R	CGGTACCGGATCC*GGGCACGGGCAGCTTGCCGG****TGG***cAAGAAGGGCACCACCTTG
	µ5	dna1822	luc2-eGFP1-u5-R	CGGTACCGGATCC*GGGCACGGGCAGCTTGCCGG****TGG***cCCTCGAAGAAGGGCACCACCTTG
	µ10	dna1823	luc2-eGFP1-u10-R	CGGTACCGGATCC*GGGCACGGGCAGCTTGCCGG****TGG***ctTTAGCCTCGAAGAAGGGCACCACCTTG
	µ20	dna1825	luc2-eGFP1-u20-R	CGGTACCGGATCC*GGGCACGGGCAGCTTGCCGG****TGG***cAaTCaACgACtTTAGCCTCGAAGAAGGGCACCACCTTG
	µ30	dna1827	luc2-eGFP1-u30-R	CGGTACCGGATCC*GGGCACGGGCAGCTTGCCGG****TGG***cCCGGTGTCCAAaTCaACgACtTTAGCCTCGAAGAAGGGCACCACCTTG
	pGLK-CMV-luc2 (µH 40, 50 bp)	dna1848	luc2-uH-F2	CGAGGCTAAaGTcGTtGAtTTGGACACCGGTAAGACACTGGGTGTGAACCAGCGCGGCGAGCTGTGCGT
		dna1847	luc2-u40plus-R2	cAGTGTCTTACCGGTGTCCAAaTCaACgACtTTAGCCTCGAAGAAGGGCACCACCTTGCCTACTGCGCCA
	Common (µH 40, 50 bp)	dna1844	eGFP1-CamccdB-R2	ACgACtTTAGCCTCGg***CCA****CCGGCAAGCTGCCCGTGCCC*GCGGCCGCGAATTCTGTCGACCTGCAGACTGGCTGTG
	µ40	dna1845	eGFP1-CamccdB-u40-F	ACCGGTAAGACACTg***CCA****CCGGCAAGCTGCCCGTGCCC*GGATCCGGTACCGAATTCGCGGCCGCATTAGGCAC
	µ50	dna1846	eGFP1-CamccdB-u50-F	ACCGGTAAGACACTgGGTGTGAACCg***CCA****CCGGCAAGCTGCCC*GTGCCCGGATCCGGTACCGAATTCGCGGCCGCATTAGGCAC

Lower-case characters indicate overhangs for BsaI cloning in SSA primers, and silent mutations in MMEJ primers. Operational sequences in MMEJ Assay primers are annotated as follows: underline, InFusion homology; italics, eGFP1 protospacer; bold italics, PAM; double underline, microhomology. For µ40 and µ50 assembly, InFusion sites were within the engineered microhomology.

Supplementary Table 7. Primers used for genotyping in this study

Gene	PCR Reaction	Primer ID#	Primer Name	Sequence	Product Size (bp)
HPRT	*HPRT1*_B mutation analysis	dna309	hHPRT1Cel-Fo	TTTCTGTAGGACTGAACGTCTTGCTC	305
		dna310	hHPRT1Cel-Rev	ATCTCACTGTAACCAAGTGAAATGAAAGC	
	*Afl*II RFLP, mutation analysis	dna1720	hHPRT-5int-8F	GAAGTTTAATGACTAAGAGGTGTTTG	619
		dna411	hHPRT1Cel-Rev2	ATTTGTCAAACCTAGCTCCAAAGG	
	5' end	dna319	HPRT1-LaF	GTGGAATTTCTGGGTCAAGGGGAAAGAG	1158
		dna804	AAVS1genoS1-2	GAGCCTAGGGCCGGGATTCTC	
	Spanning (non-targeted allele)	dna319	HPRT1-LaF	GTGGAATTTCTGGGTCAAGGGGAAAGAG	1868
		dna383	HPRT1-RaR2	AGGCGAGTTTCTACAAAGATGGACAGG	
	3' end (KW668)	dna930	TKseq	CCGCGCACCTGGTGCATGAC	2158
		dna383	HPRT1-RaR2	AGGCGAGTTTCTACAAAGATGGACAGG	
	3' end (KW836, KW838)	dna123	mCherry-F	CCGTAATGCAGAAGAAGACCAT	1748
		dna383	HPRT1-RaR2	AGGCGAGTTTCTACAAAGATGGACAGG	
APRT	T7E1, *Acc*65I RFLP	dna1711	hAPRT-T7F5	GTCGTGGATGATCTGCTGG	461
		dna1712	hAPRT-T7R5	TGCCCAAGGCTGATATTTCC	
	5' end	dna1728	hAPRT-e1e2-F2	CTTCCGGCGACGGATGCC	2287
		dna804	T2A-puroJ	GAGCCTAGGGCCGGGATTCTC	
	Spanning (non-targeted allele)	dna1796	SNP-rs3826074-F	TCCTCCATTTCCACCTTCCCTA	4020
		dna1865	hAPRT-HAR2	GCTTGCTCCCCTAGAAGATG	
	3' end	dna116	rBgSp1b	ATGAACAAAGGTGGCTATAAAGAGGTCATC	876
		dna1865	hAPRT-HAR2	GCTTGCTCCCCTAGAAGATG	

Supplementary Table 8. Primers used for sequencing in this study

Template	Application	Primer ID#	Primer Name	Sequence
HPRT	Targeted 5' arm junctions	dna319	HPRT1-LaF	GTGGAATTTCTGGGTCAAGGGGAAAGAG
		dna1733	HPRT-seq2	CCTTTGCCCTCATGTTTCAT
		dna309	hHPRT1Cel-Fo	TTTCTGTAGGACTGAACGTCTTGCTC
	Targeted 3' arm junctions	dna116	rBgSp1b	ATGAACAAAGGTGGCTATAAAGAGGTCATC
		dna117	rBgSp2c	CCCAGTCATAGCTGTCCCTCTTCTCTTATG
APRT	Targeted 5' arm junctions	dna1726	hAPRT-5int-1R	AGATCATCCACGACGACCAC
	Targeted 3' arm junctions	dna116	rBgSp1b	ATGAACAAAGGTGGCTATAAAGAGGTCATC
		dna1725	hAPRT-3int-10F	GGAAATATCAGCCTTGGGCA
	Spanning, Targeted 5' arm junctions	dna1711	hAPRT-T7F5	GTCGTGGATGATCTGCTGG
		dna1692	APRT-HAF	ACTCCTGTCACTTACCCTGA
TOPO Products	Universal PCR and sequencing	T3		ATTAACCCTCACTAAAGGGA
		T7		TAATACGACTCACTATAGGG

Supplementary Table 9. Primers used for exon genotyping in this study

Gene	Exon			Fwd Primer		Amplicon Size
	no.	ENSEMBL exon ID	Length	Name	Sequence	
HPRT	1	ENSE00001913528	186	dna1871	CAGGGAGCCCTCTGAATAGGA	536
				dna1872	GTGACGTAAAGCCGAACCC	
	2	ENSE00003489858	107	dna1873	TAGTAGAGACGGGATTTCACC	466
				dna1874	AGAACAGCTGCTGATGTTTGA	
	3	ENSE00003623041	184	dna1875	TTGGTGTGGAAGTTTAATGACTAAG	385
				dna1876	ATCTCACTGTAACCAAGTGAAATG	
	4	ENSE00003674574	66	dna1877	TCTAGTCATTCATTTCAGGAAACCT	339
				dna1878	ATTGATTGAAAGCACACTGTTACT	
	5	ENSE00003522510	18	dna1879	AGCAGATGGGCCACTTGTTA	252
				dna1880	TGGCTTACCTTTAGGATGGTG	
	6	ENSE00003576599	83	dna1881	GGGCCAGATGATATAGATTCCA	332
				dna1882	TGACAGTTGAAAACATTTATCCTTA	
	7, 8	ENSE00003676328, ENSE00003495603	47, 77	dna1883	TGCTGCCCCTTCCTAGTAATC	651
				dna1884	GCCAGGTTCCAGTTCTAAGGA	
	9	ENSE00001904310	639	dna1885	TGTGATAGACTACTGCTTTGTTTTC	1019
				dna1886	CCGCCAACCCATTCTACC	
APRT	1 + 2	ENSE00002586104, ENSE00001503918	125, 107	dna1728	CTTCCGGCGACGGATGCC	640
				dna1729	CTCAATCTCACAACCCTTCCCG	
	3, 4, 5	ENSE00001503917, ENSE00003473485, ENSE00002584924	134, 79, 143	dna1740	CATGGGGAGAGGAAGGTGT	1255
				dna1741	GTACAGGTGCCAGCTTCTCC	

Supplementary Table 10. Primers used for Southern blot probe preparation in this study

Gene	Probe	Primer ID#	Primer Name	Sequence	Product Size (bp)
HPRT	HPRT-B (5' External)	dna1718	hHPRT-5ext-4F	GCTGAGGATTTGGAAAGGGT	475
		dna1719	hHPRT-5ext-4R	GCCAGACATACAATGCAAGC	
APRT	APRT (5' Internal)	dna1692	hAPRT-HAF	ACTCCTGTCACTTACCCTGA	496
		dna1726	hAPRT-5int-1R	AGATCATCCACGACGACCAC	
Common	mCherry	dna1737	mCh-probeF	GTTCATGTACGGCTCCAAGG	505
		dna062	UniFruitR	TTACTTGTACAGCTCGTCCATGC	

Supplementary References

1. Ran, F.A. et al. Genome engineering using the CRISPR-Cas9 system. *Nature protocols* **8**, 2281-2308 (2013).

2. Fu, Y. et al. High-frequency off-target mutagenesis induced by CRISPR-Cas nucleases in human cells. *Nature biotechnology* **31**, 822-826 (2013).

3. Oceguera-Yanez, F. et al. Engineering the AAVS1 locus for consistent and scalable transgene expression in human iPSCs and their differentiated derivatives. *Methods* **101**, 43-55 (2016).
